# In vitro-in vivo correlations of pulmonary inflammogenicity and genotoxicity of MWCNT

**DOI:** 10.1186/s12989-021-00413-2

**Published:** 2021-07-23

**Authors:** Emilio Di Ianni, Johanna Samulin Erdem, Peter Møller, Nicklas Mønster Sahlgren, Sarah Søs Poulsen, Kristina Bram Knudsen, Shan Zienolddiny, Anne Thoustrup Saber, Håkan Wallin, Ulla Vogel, Nicklas Raun Jacobsen

**Affiliations:** 1 National Research Centre for the Working Environment, DK-2100 Copenhagen, Copenhagen, Denmark; 2 National Institute of Occupational Health, Oslo, Norway; 3 Department of Public Health, University of Copenhagen, Copenhagen, Denmark; 4 Evaxion Biotech, DK-1260 Copenhagen, Denmark; 5 DTU Food, Technical University of Denmark, DK-2800 Kgs. Lyngby, Denmark

**Keywords:** Multiwalled carbon nanotube, In vitro-in vivo correlation, Respiratory toxicity, In vitro alternative, Nanomaterial

## Abstract

**Background:**

Multi-walled carbon nanotubes (MWCNT) have received attention due to extraordinary properties, resulting in concerns for occupational health and safety. Costs and ethical concerns of animal testing drive a need for in vitro models with predictive power in respiratory toxicity. The aim of this study was to assess pro-inflammatory response (*Interleukin-8 expression, IL-8*) and genotoxicity (DNA strand breaks) caused by MWCNT with different physicochemical properties in different pulmonary cell models and correlate these to previously published in vivo data. Seven MWCNT were selected; two long/thick (NRCWE-006/Mitsui-7 and NM-401), two short/thin (NM-400 and NM-403), a pristine (NRCWE-040) and two surface modified; hydroxylated (NRCWE-041) and carboxylated (NRCWE-042). Carbon black Printex90 (CB) was included as benchmark material. Human alveolar epithelial cells (A549) and monocyte-derived macrophages (THP-1a) were exposed to nanomaterials (NM) in submerged conditions, and two materials (NM-400 and NM-401) in co-cultures of A549/THP-1a and lung fibroblasts (WI-38) in an air-liquid interface (ALI) system. Effective doses were quantified by thermo-gravimetric-mass spectrometry analysis (TGA-MS). To compare genotoxicity in vitro and in vivo, we developed a scoring system based on a categorization of effects into standard deviation (SD) units (< 1, 1, 2, 3 or 4 standard deviation increases) for the increasing genotoxicity.

**Results:**

Effective doses were shown to be 25 to 53%, and 21 to 57% of the doses administered to A549 and THP-1a, respectively. In submerged conditions (A549 and THP-1a cells), all NM induced dose-dependent *IL-8* expression. NM-401 and NRCWE-006 caused the strongest pro-inflammatory response. In the ALI-exposed co-culture, only NM-401 caused increased *IL-8* expression, and no DNA strand breaks were observed. Strong correlations were found between in vitro and in vivo inflammation when doses were normalized by surface area (also proxy for diameter and length). Significantly increased DNA damage was found for all MWCNT in THP-1a cells, and for short MWCNT in A549 cells. A concordance in genotoxicity of 83% was obtained between THP-1a cells and broncho-alveolar lavaged (BAL) cells.

**Conclusion:**

This study shows correlations of pro-inflammatory potential in A549 and THP-1a cells with neutrophil influx in mice, and concordance in genotoxic response between THP-1a cells and BAL cells, for seven MWCNT.

**Supplementary Information:**

The online version contains supplementary material available at 10.1186/s12989-021-00413-2.

## Background

Multi-walled carbon nanotubes (MWCNT) are high-aspect ratio nanomaterials (NM) with electrical, mechanical and thermal properties that make them promising for various industrial applications [1, 2]. The increased production of MWCNT has raised concern for workers health. When inhaled, MWCNT are biopersistent in the lung and, similarly to asbestos, have been shown to induce chronic inflammation, fibrosis and cancer in rats following chronic inhalation [3–6]. Notably, one long MWCNT (MWCNT-7) was classified as possibly carcinogenic to humans by IARC [7], based on animal data [6, 8, 9]. Other MWCNT were not classified as carcinogens by IARC because of a knowledge gap between in vivo results on cancer outcome and inconclusive observations on mechanisms of action in cell cultures and animal models [10].

Several in vivo studies have determined how different physicochemical properties of MWCNT affect the lungs of mice or rats exposed via inhalation or intra-tracheal instillation [11, 12, 15, 17–22]. These studies provide valuable information on the distribution and local or systemic effects of MWCNT. However, testing the multitude of new NM on animals is challenging due to time, cost, and ethical concerns. Therefore, in vitro cell models are being developed to replace, reduce, and refine the use of animals. While many in vitro studies are published yearly to assess the pulmonary toxicity of NM, including MWCNT, there is still a lack of studies showing how the effects in these models correlate with the in vivo responses [23]*.* As a result, in vitro models are presently not used for risk assessment, or regulation, of MWCNT and other NM, without animal data. Predictive in vitro models are urgently needed.

Our group has previously investigated the respiratory in vivo toxicity of a vast number of MWCNT with diverse physicochemical properties, including length, diameter, shape, surface modification and metal impurities. Several end-points including pulmonary inflammation and DNA damage were explored [11, 15, 17, 22, 24–31]. The aim of this study was to assess MWCNT-induced pro-inflammatory responses and levels of DNA strand breaks in cell culture models, and assess how the in vitro results correspond with MWCNT-induced effects previously observed in vivo. The MWCNT physicochemical characterization and our previously published in vivo data guided the selection of MWCNT to be tested in vitro. We selected 7 MWCNT with different diameter, length, specific surface area, level of surface oxidation and metal impurities. Carbon black (CB) Printex 90 was included as benchmark NM, to increase comparability with previous studies [11, 12, 17, 29, 31–33]. We tested these NM on two human derived mono-cultures; type II alveolar epithelial cells (A549) and activated THP-1 macrophages (THP1-a) in submerged conditions. These cell lines have been used extensively in respiratory toxicology, with easy access due to low biosafety level, thus allowing for comparisons with other studies. Furthermore, previous studies have identified these as useful for pulmonary toxicity testing and relevant to humans [16, 23, 34]. Co-cultures of A549, THP-1a and WI-38 (human fibroblasts) cells were exposed to one short and one long MWCNT using an air-liquid interface system, to assess toxicity in a more complex model than mono-cultures exposed in submerged conditions. To assess the pro-inflammatory response and correlate to neutrophil influx in vivo, we quantified gene expression of the neutrophil chemo-attractant interleukin-8 (*IL-8,* also known as C-X-C motif chemokine ligand 8 (*CXCL8*)). *IL-8* is an early pro-inflammatory biomarker and highly expressed during NM-induced inflammation in both human and murine cells (mouse homologue gene is *Cxcl-1*), as a result of the NF-kβ inflammatory pathway [35–38]. *IL-8* mechanistic relevance has been further indicated in Adverse Outcome Pathways frameworks [26]. In addition, we have previously found *Cxcl-1* to be induced in a dose-dependent manner in lungs of mice following exposure to MWCNT [12, 22, 31]. Damage to DNA was quantified as levels of DNA strand breaks by the alkaline comet assay. The obtained endpoints related to pro-inflammatory response and genotoxicity in vitro were correlated to the previously generated in vivo data.

## Methods

### Nanomaterials

Seven MWCNT were selected for the in vitro study (Table 1). Printex 90 was included as a benchmark NM to increase comparability with the materials that were previously tested in our group [11, 12, 17, 29, 31–33]. The rationale of the material selection was to include two long and thick (NRCWE-006 and NM-401), two short and thin (NM-400 and NM-403), and three short fibers (Cheap Tubes) with similar physical properties, but with different levels of surface modification; pristine (NRCWE-040), hydroxylated (NRCWE-041) and carboxylated (NRCWE-042) functionalized MWCNT. Our group has previously characterized these materials thoroughly [11, 14, 32] (Table 1 and Table S1). The pristine (NRCWE-040) and surface modified MWCNT (NRCWE-041 and -042) were purchased from Cheap Tubes (Battleboro, VT, USA). Carbon black Printex 90 was a gift from Degussa-Hüls (today Evonik), Frankfurt, Germany. NRCWE-006 (MWCNT-XNRI-7) was a gift from Mitsui, Tokyo, Japan. NM-400, NM-401 and NM-403 were obtained from the nanomaterial repository at the European Joint Research Centre, Ispra, Italy. All materials were tested here in vitro and previously in vivo, with one exception; NRCWE-026 was tested in vivo and NM-400 in vitro. NRCWE-026 is the same product as NM-400, but from a different production batch. The physico-chemical characterization of these materials indicated that NRCWE-026 contains more Al than NM-400 [14, 39].

**Table 1:** Physicochemical properties of the NMs included in this study

Nanomaterial	Length nm	Diameter nm	BET m^**2**^/g	OH mmol/g	A549 Z-average F12 (nm)	A549 Effective dose^**5**^	A549 Percent of administered dose (%)	THP-1a Z-average RPMI (nm)	THP-1a Effective dose^**e**^	THP-1a Percent of administered dose (%)
NRCWE-040^a^	519	22	150	0.35	199 ± 23	74	46	266 ± 26	42	52
NRCWE-041^a^	1005	27	152	1.69	215 ± 15	85	53	283 ± 27	46	57
NRCWE-42^a^	723	30	141	4.09	178 ± 10	40	25	276 ± 14	30	37
NRCWE-006^b^	5730	74	26	0.08	631 ± 183	40	25	917 ± 80	17	21
NM-400^b,d^	847	11	254	0.79	307 ± 94	42	26	223 ± 60	29	36
NM-401^b,d^	4048	67	18	0.03	760 ± 122	74	46	978 ± 74	31	39
NM-403^b^	443	12	135	0.19	245 ± 39	54	34	285 ± 25	36	45
Printex 90^c^	–	14	338	–	125 ± 4	40	25	168 ± 3	38	47

The nanomaterials were previously characterized. Data are extracted from ^a^Poulsen et al. 2016, ^b^Jackson et al., 2015 and ^c^Jacobsen et al., 2008. ^d^The hydrodynamic diameter of NM-400 and NM-401 dispersed in MilliQ-Water for cell exposure in ALI system was 205 ± 7 and 701 ± 11 nm, respectively. ^e^ Effective dose quantified by TGA-MS after 24 h incubation as described in Methods. – Not detected

### Cell cultures

Alveolar epithelial cells (A549), monocytic THP-1 cells and fibroblasts (WI-38) were purchased from the American Type Culture Collection (ATCC; Rockville, MD, US). A549 cells were cultured in Hams F-12 (F-12; Gibco, Carlsbad, CA, #31765035) cell culture medium supplemented with 10% fetal bovine serum (FBS; Gibco, Carlsbad, CA, #10106–169), and 1% penicillin/streptomycin (P/S; Gibco, Carlsbad, CA, #15140–122). The cell models were selected based on a screening of the literature on in vivo-in vitro correlation on pulmonary end-points such as inflammation and histology. In addition, we considered cell types with biosafety level 1. A549 cells were cultured in culture flasks at 37 °C and 5% CO_2_, and trypsinized and subcultured when 70% cell confluence was reached (~ 2 times a week). Monocytic THP-1 cells were cultured in suspension in RPMI-1640 (RPMI; Gibco, Carlsbad, CA, #72400047) containing Glutamax, supplemented with 10% FBS and 1% P/S. THP-1 cells were cultured at density of 3-9 × 10^5^ cells/ml complete culture medium at 37 °C and 5% CO_2_, and sub-cultured three times a week. WI-38 cells were cultured in Eagle minimum essential medium (Corning, NY, USA, #10–009-cv) supplemented with 10% FBS and 1% P/S. WI-38 cells were cultured in culture flaks at 37 °C and 5% CO_2_, trypsinized when cells reached 70% confluence. To prevent phenotypic changes of cell lines at high passage numbers, each cell line batch was cultured for 10–13 passages and then discarded. Twenty-four hour prior to exposure, A549 cells were seeded at a density of 2 × 10^5^ cells/ml in Nunc 12-well plates (3.5 cm^2^; Biotech Line, Slangerup, Denmark) in 2 ml. Before exposure, the medium was removed and fresh medium added. Monocytic THP-1 cells were diluted to 3 × 10^5^ cells/ml in 2 ml and differentiated into macrophage-like cells (THP-1a) with 10 ng/ml of phorbol-12-myristate-13-acetate (PMA, Sigma Aldrich, Sweden), for 48 h, in 12-well plates. Prior to NM exposure, the medium was removed and fresh medium added.

### Particle suspensions and exposure in submerged conditions or air-liquid interface

All NM were weighed (17–20 mg) in glass vials, to which 5–6 ml of complete cell culture medium (F12 or RPMI-1640 for A549 or THP-1a cells, respectively, containing 10% FBS and 1% P/S) was added to obtain a final particle suspension of 3.34 mg/ml. The particle suspensions were sonicated with a Branson Sonifier for 16 min, at 10% amplitude, and 10s impulses. During sonication, the vials containing NM suspensions were kept in a water bath with ice. The stock suspensions were then diluted for exposure under submerged conditions; A549 cells were exposed to 10, 40 or 160 μg/ml (2.8, 11.4, and 45.7 μg/cm^2^); THP-1a cells were exposed to 10, 40 or 80 μg/ml (2.8, 11.4, and 22.8 μg/cm^2^). Complete cell culture medium was used as vehicle control (reported as 0 μg/ml). All submerged exposures were replicated in at least three independent experiments.

The effects of a smaller subgroup of MWCNT (NM-400 and NM-401) were assessed also in a more complex in vitro model. Namely, pro-inflammatory response and genotoxicity were assessed in co-cultures of A549 and THP-1a (apical side of inserts) exposed in an ALI system, and in contact with WI-38 cells (basolateral side of insert), as described in details in the Supplement.

### Dosimetry

Effective doses of MWCNT and CB on A549 and THP-1a cells exposed under submerged conditions were determined by thermo-gravimetric-mass spectrometry (TGA-MS) analysis. A549 (400.000 cells) and THP-1a (600.000 cells) were seeded in 12-well plates and exposed to 160 and 80 μg, respectively, of each NM. Following 24 h exposure, the medium was gently removed and cells harvested by vigorously washing with 500 μl of PBS/well and scraping with the tip of a pipette. The samples from all 12 wells were combined and centrifuged (20.000G for 30 min) to obtain a pellet of NM and cells. We exposed the cells to the highest doses used in the in vitro toxicity assessment, and in 12 wells, to ensure a total mass of samples above the limit of detection of the TGA (80 μg). The precipitated pellets were placed into a crucible for the TGA-MS analysis, which was based on a NANoREG protocol as previously described [40]. Briefly, the TGA-MS method was carried out on Netzsch STA 449F3 (Netzsch-Gerätebau GmbH, Selb, Germany) coupled with gas capillary transfer line to QMS D Aëolos mass spectrometer (Netzsch-Gerätebau GmbH, Selb, Germany). The method is a one step program starting at 30 °C and with a heating rate of 2.5 °C /min until 800 °C is reached. During the heating program gas molecules are transferred to the mass spectrometer. The TGA-MS running program has a duration of 308 min. The carbon content stemming from NM was quantified from the mass loss between 450 and 700 °C, temperatures at which the MWCNT or CB would burn and emit CO_2_. The mass loss was used to calculate elemental carbon mass and used as effective doses to which the cells were exposed. The variation in the method was estimated through three independent experiments with NRCWE-006. The obtained variation includes everything from cell seeding, NM preparation, exposure and harvest as well as the TGA-MS analysis. The averaged mass detected from three independent experiments was 0.48 ± 0.06 mg, which is 25% of the total 1.92 mg NRCWE-006 administered in 12 wells (160 μg/well) (Fig. S1).

### Microscopy

Uptake of NM in cells was assessed by bright field microscopy. After exposure of A549 and THP-1a cells to 40 μg/ml in submerged conditions, cells were washed once, trypsinized and resuspended as single cell suspension as described elsewhere [41]. Briefly, 50 μL of the cell resuspension in HAM-F12 or RPMI-1640 medium was transferred to a microscope slide and centrifuged at 10,000 rpm for 4 min using a Cytofuge 2 apparatus (StatSpin, Bie and Berntsen, Rødovre, Denmark). Cells were fixed by the addition of 96% ethanol and stained with May-Grüunwald-Giemsa stain. NM uptake was qualitatively assessed as black matter inside the cytosol. Cells were imaged at 100x magnification.

### Toxicity

#### Viability

Following 6 or 24 h of cell exposure to NM in submerged conditions, cell medium was discarded and cells (A549 or THP-1a) were washed twice with PBS. Two hundred μl of trypsin was added to each well, the plates were incubated for 4 min after which 300 μl of cell culture medium was added. Cells were gently pipetted to obtain a single cell suspension and further detach those cells that were still attached to the cell culture plate. Cell viability was measured by NucleCounter (ChemoMetec A/S, Allerød, Denmark). Similarly, 24 h after exposure in the ALI system, A549 and THP-1a co-cultures were trypsinized, and the cell viability assessed by NucleoCounter (since at 24 h over 98% of the cells were viable, viability was not assessed after 6 h). Control experiments were performed to assess a possible interference of MWCNT with the acridine orange dye used in the NucleoCounter. Seven hundred thousand THP-1 cells/ml were suspended in 12-well plates. Zero, 20, 40, 80, 160, and 320 μg/ml of a long (NM-401) or a short (NM-403) suspended MWCNT were added to the cell suspensions, after which cells were counted by NucleoCounter. A decrease in total counted cells was only observed with concentrations of NM-403 above 80 μg/ml (a reduction of ≥30%); no change was observed with NM-401 concentrations up to 320 μg/ml. Although the tested NM concentrations were high compared to effective doses, no impact of the interference on cell viability measurement was observed (Table S4C).

#### Interleukin-8 expression

Total RNA was isolated from harvested cell samples with Macherey-Nagel® NucleoSpin 96-well RNA Core Kit (Macherey-Nagel, Düren, Germany), as recommended by the manufacturer. RNA concentration and purity were determined by NanoDrop 2000C (ThermoFisher, Wilmington, USA) according to the manufacturer’s instructions. Similar RNA quantity and purity across samples indicated that the MWCNT and CB did not affect RNA purification in the used dose range. cDNA was synthesized with Taqman® reverse transcription reagents (Applied Biosystems, USA), as recommended by the manufacturer. The quantitative PCR was performed on an (PE Biosystems, Foster City, CA, USA) “with” ViiA7 real-time PCR system, using Universal Mastermix (Applied Biosystems, Naerum, Denmark) and pre-developed primers for IL-8 (part. no. 4327042F) and r18S (part. no.4310881E) as reference gene from Applied Biosystems. The samples were run in triplicate. The mRNA levels were then further normalized to controls (0 μg/ml of tested NM) to take the day to day variation of the assay into account.

#### DNA damage

DNA strand break levels were quantified by the comet assay described previously [42]. Following cell exposure to NM, for 6 or 24 h, cell media were discarded, and cells (A549 or THP-1a) were washed twice with PBS. Two hundred μl of trypsin was added to each well, the plates were incubated for 4 min after which 300 μl of freezing medium (80%FBS and 20% DMSO) was added. Cells were stored at − 80 °C. Frozen cell samples were thawed quickly at 37 °C and suspended in agarose at 37 °C with final agarose concentration of 0.7%. Cells were embedded on 20-well Trevigen CometSlidesTM (30 mL per well). Slides were cooled and placed in lysis buffer overnight at 4 °C, after which these were placed in electrophoresis buffer for 40-min alkaline treatment. Electrophoresis was run with 70 ml/min circulation (5%) of the solution for 25 min with applied voltage at 38 V (1.15 V/cm in the whole electrophoresis tank) and current of 294 mA. The slides were neutralized in Tris buffer (2 × 5 min), fixed in ethanol for 5 min and dried on a warm plate at 45 °C for 15 min. Cells on slides were stained in 40 ml/slide bath with TE buffered SYBR Green fluorescent stain for 30 min, dried at 37 °C for 10 min after which UV-filter and cover slips were placed on slides. DNA damage was analyzed using the IMSTAR Pathfinder system. The results are presented as averaged % tail of DNA of all cells scored on each Trevigen CometSlide. All slides included A549 cells exposed to PBS or 45 mM H_2_O_2_ as negative and positive controls for the electrophoresis [42] (DNA % tail = 3.2 ± 2.3 and 20.5 ± 1.8, for negative and positive controls, respectively).

### In vivo

All materials tested in the present study have been previously tested in vivo. We analyzed the NM-induced effects in broncho-alveolar lavage (BAL) cells in addition to lung tissue in all our studies, as BAL cells are located on the lung epithelium with an exposure comparable to the lung epithelium at the alveolar surface. The in vivo toxicity data have been published previously [11, 12, 22], except inflammation and genotoxicity in mice exposed to NM-403, and pulmonary genotoxicity in mice exposed to NRCWE-006 which have been included in this paper to increase the dataset for in vitro-in vivo comparisons. The animal studies were performed as described in Poulsen et al., 2017 [13]. Briefly, mice were exposed via intra-tracheal instillation of 6, 18, and 54 μg of NM-403 and 18, 54 and 162 μg of NRCWE-006 and followed for one day, after which they were terminated. BAL cells and lung tissue were collected and examined as in Poulsen et al., 2017 [13]. Analysis of neutrophil infiltrates in BAL fluid and DNA strand breaks levels in BAL cells and lung tissue were performed as described in Poulsen et al., 2016 [11]. The study complied with the EC Directive 86/609/ EEC on the use of animals for experiments and they were approved by the Danish ‘Animal Experiments Inspectorate’ under the Ministry of Justice (permission 2010/561–1779) and by the local ethical committee for animal research.

### Statistics

The data on DNA damage levels displayed day to day variation. Thus, DNA damage levels were assessed using one-way ANOVA (effect of treatment) with a block adjusted for day to day variation in background levels of DNA damage. Inflammation (log-fold increase with respect to controls) was assessed by one-way ANOVA, with dose as independent variable. Both analyses were followed by post-hoc Tukey-type multiple comparison test for effects showing statistical significance in the overall ANOVA test. Statistical significance was tested at *P* < 0.05 level. The statistical analyses were performed in R (The R Project for Statistical Computing version 3.5.3). The package *ggplot2* was implemented in R for plotting of data [43]. The Pearson Correlations and all multiple regression analyses were performed in SAS version 9.4 (SAS Institute Inc., Cary, NC, USA).

#### Correlation of in vitro-in vivo inflammation

In order to assess in vitro*-*in vivo correlation of inflammation of MWCNT, we determined the inflammatory effect of MWCNT in vivo and in vitro and assessed the correlation between the models. Neutrophil influx quantified in four previously published in vivo studies with MWCNT were used for the analysis [11, 12, 22] (NM-403 in Table S2). The controls from the in vivo studies were pooled, as no statistically significant variation was seen across studies. Linear regression models were used to assess the dependency of inflammation on dose (mass or surface area) and the MWCNT type. Doses were normalized to both mass and MWCNT surface area since MWCNT surface area was correlated to neutrophil influx following mice exposure via inhalation and instillation [11, 15, 22]. The slopes from the regression analyses based on surface area were used to describe difference in MWCNT inflammatory potential in vivo. Similarly, regression analyses were performed for the MWCNT-induced pro-inflammatory response (*IL-8* expression) in A549 and THP-1a cells following 6 and 24 h exposure, and slopes used to determine MWCNT pro-inflammatory potential in vitro with doses as mass and surface area (proxy of diameter and length). Finally, the NM pro-inflammatory potential in vitro and inflammatory potential in vivo were correlated with Pearson’s correlations.

#### Scoring system for comparing in vitro and in vivo genotoxicity

In order to assess in vitro-in vivo correlations of NM-induced DNA damage, we chose to include both statistical significant effects as well as larger non-significant effects. The statistical analysis was conducted as a traditional ANOVA test, whereas the developed scoring system was based on categorization of effects normalized to standard deviation units. The output is similar to the standardized mean difference that quantitates the difference between exposed and controls in terms of SD differences, which we have previously used to assess differences in genotoxicity on different scales [44, 45]. Such standardization would make in vitro-in vivo comparisons of genotoxicity more robust, given the different in vitro and in vivo experimental procedures, and different dose-response relationships; in vivo exposure to NM led to bell-shaped dose-response relationships. To assign the categories, all the controls in e.g. A549 cell experiments were pooled together. The difference between exposed and controls (delta value = exposed – control) was compared to the SD in the controls. As a conservative approach we assumed that differences larger than 2*SD are “true” genotoxic effects. We have used 2*SD as limit because it is the traditional cut-off of “outliers”, meaning that the effect in the exposed group is outside the range of random variation in unexposed cells or animals. On the genotoxic scale, each level of difference between the exposed and controls corresponds to one extra SD unit (for simplicity we have highlighted effects higher than 4*SD with the same color in the heat map) (Table S3). In the heat map, we have also outlined the outcome of statistical testing, with stars indicating statistical significance levels from the ANOVA test. From the heat map, concordance in genotoxicity between in vitro and in vivo was assessed by comparing positive and negative genotoxicity. With this system, we were able to give weight to the genotoxic effects (2-fold or larger increase), which were not possible to identify statistically, due to the variation in the data.

## Results

### Nanomaterials characterization

The physicochemical properties of the MWCNT tested here were previously characterized [11, 17], and are shown in Table 1 and Table S1, including NM dimensions, specific surface area (BET), and levels of surface oxidation (OH).

In addition, Table 1 reports the hydrodynamic diameters determined by dynamic light scattering (DLS) after MWCNT were suspended in complete cell culture media (Hams-F-12 or RPMI-1640 cell media containing 10% FBS and 1% P/S) or milliQ-water. NM-401 and NRCWE-006 formed the largest agglomerates in both cell media (diameter 600–1000 nm), whereas the other NM agglomerates resulted in hydrodynamic diameters between 100 and 300 nm in both cell media. Although the size ranges of suspended particles overlapped in the two media, we generally saw that the mean agglomerate size was increased by 50–250 nm in RPMI-1640 medium compared to Hams-F-12. Suspensions of NM-401 and NM-400 in milliQ water containing 0.05% BSA resulted in hydrodynamic diameters of 700 and 205 nm, after 40 μm filtration.

#### Dosimetry

We determined the effective doses of MWCNT and CB in both A549 and THP-1a cells following 24 h incubation in submerged conditions by TGA-MS (Table 1). The effective doses in A549 cells spanned from 40 to 85 μg (25 to 53% of the administered doses). Largest effective dose was observed for the hydroxylated MWCNT (NRCWE-041), followed by NM-401, NRCWE-040, NM-403, and following NRCWE-042, NM-400, and CB. In THP-1a cells, effective doses spanned between 17 and 46 μg (21 to 57% of the administered dose). Largest effective dose was observed for NRCWE-041, followed by NRCWE-040, CB, NM-403, NM-401, NRCWE-042, NM-400 and NRCWE-006.

### Toxicity

#### Viability

**Fig. 1:**
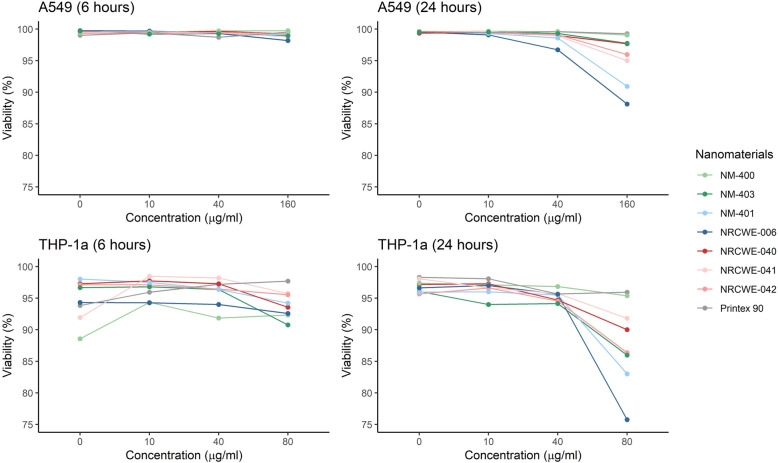
Viability of A549 (top) and THP-1a cells (bottom). Cells were exposed to carbon nanotubes for 6 h and 24 h. The results represent the mean of three independent experiments. Error bars were omitted for graphical reasons; the full data table is available in Table S4

The tested NM concentrations did not significantly affect viability of A549 or THP-1a cells (Fig. 1). For the epithelial cells (A549), viability was above 88% across the tested concentrations (10–160 μg/ml). For the macrophages (THP-1a), a slight but insignificant decrease of ~ 20%, was observed with the larger MWCNT (NRCWE-006) (Table S4A). No decrease in cell viability was observed in the co-culture exposed to high dose and low dose of NM-400 or NM-401 in the ALI system (Table S4B).

#### Interleukin-8 expression

**Fig. 2:**
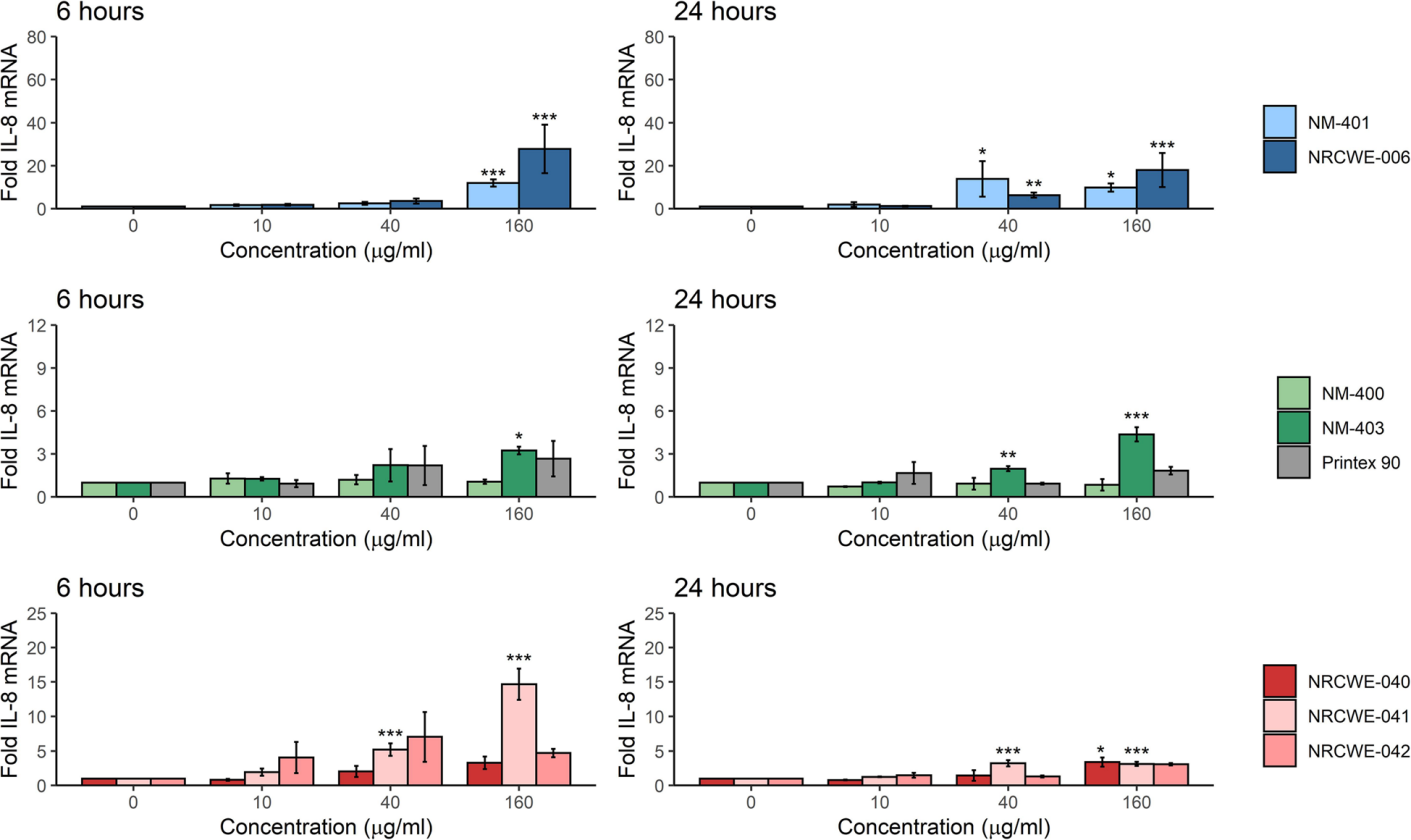
Fold increase of *IL-8* mRNA levels (Cxcl8) in A549. Cells were exposed to NM for 6 or 24 *h. IL-8* mRNA levels were normalized to 18S and then to vehicle control. The values are mean ± SEM of minimum three independent experiments. *: *p*≤0.05, **: *p*≤0.01, ***: *p*≤0.001

**Fig. 3:**
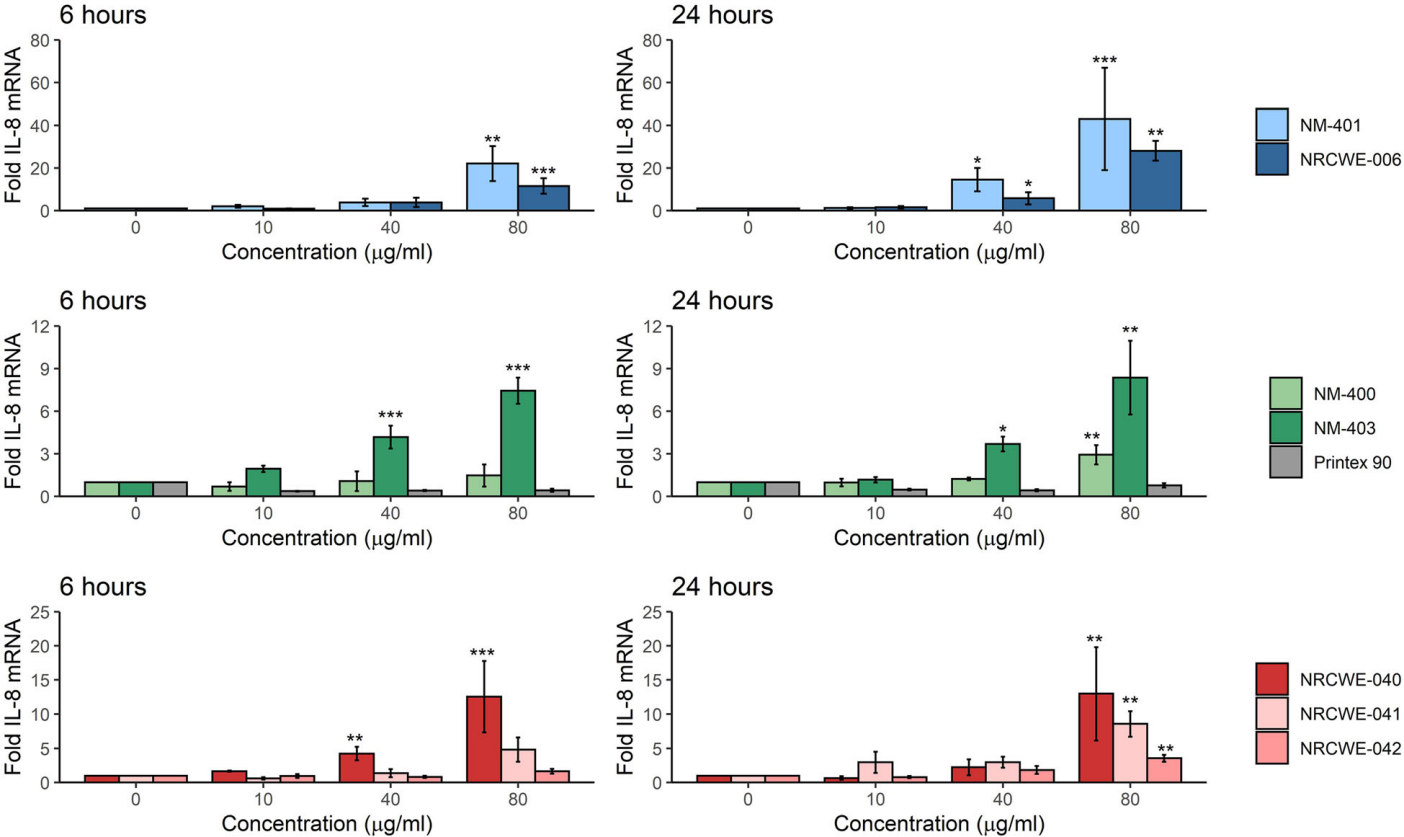
Fold increase of *IL-8* mRNA levels (Cxcl8) in THP-1a cells. Cells were exposed to NM for 6 or 24 *h. IL-8* mRNA levels were normalized to 18S and then to vehicle control. The values are mean ± SEM of minimum three independent experiments. *: *p*≤0,05, **: *p*≤0.01, ***: *p*≤0.001

The pro-inflammatory response was assessed by analysis of *IL-8* expression levels by RT-qPCR in exposed submerged mono-cultures of A549 and THP-1a cells. The choice of this pro-inflammatory marker was based on previous evidence that *IL-8* is expressed in vitro and in vivo (homologue *Cxcl-1*) in the NM-caused inflammatory response [12, 22, 26, 31]. In addition, the induction of this gene has a large dynamic range and it is highly correlated to IL-8 protein levels in A549 cells [46]. In A549 cells (Fig. 2), 160 μg/ml of long MWCNT induced significant, 30- and 18-fold increase of *IL-8* (NRCWE-006 and NM-401, respectively) after 6 h exposure. After 24 h, significant increases were observed in the cells exposed to both 40 and 160 μg/ml. A significant increase in *IL-8* was also observed in A549 cells exposed to 160 μg/ml of NM-403 for 6 h (3-fold), and to 40 and 160 μg/ml after 24 h (2.8- and 3.5-fold, respectively). The hydroxylated MWCNT (NRCWE-041) appeared to induce a higher *IL-8* expression than the carboxylated (NRCWE-042) and pristine (NRCWE-040) MWCNT after 6 h, with significant 5-fold- and 14-fold increases in A549 cells (40 and 160 μg/ml, respectively). At 24 h, these levels were decreased, but remained significantly increased (4-fold increase with 40 and 160 μg/ml). Similarly, a significant increase in *IL-8* expression was observed also in A549 cells exposed to the pristine NRCWE-040 after 24 h. A dose response relationship was observed in *IL-8* gene expression in A549 cells exposed to CB, however the levels were below two-fold increase at any time and not statistically significant. No statistically significant change was seen in A549 cells exposed to NM-400 at any time point. Concentration-response relationship of *IL-8* expression levels was observed for THP-1a cells exposed to all MWCNT (Fig. 3). Eighty μg/ml of NM-401 and NRCWE-006 induced a significant 20- and 18-fold increase in *IL-8* expression at 6 h, respectively. After 24 h, the *IL-8* expression in THP-1a cells exposed to these particles increased; 40 and 80 μg/ml of NM-401 induced statistically significant 19- and 41-fold increases, respectively, while 40 and 80 μg/ml of NRCWE-006 induced statistically significant 9- and 35-fold increase, respectively. Forty and 80 μg/ml of NM-403 increased the *IL-8* expression in THP-1a cells at 6 and 24 h, with 4- and 8-fold increase, with respect to controls. After 6 h exposure, 40 and 80 μg/ml of the pristine MWCNT (NRCWE-040) induced significantly higher *IL-8* expression in THP-1a cells (5- and 11-fold, respectively), as compared to control and to the surfaced modified MWCNT (NRCWE-041 and NRCWE-042). After 24 h, 80 μg/ml of NRCWE-040, NRCWE-041, and NRCWE-042 induced a significantly increased *IL-8* expression level in exposed THP-1a cells (12-, 9-, and 4-fold increase). NM-400 induced a significant increase of *IL-8* expression levels in THP-1a cells exposed to 80 μg/ml after 24 h. No change was seen in THP-1a exposed to CB at any time-point.

Person’s correlations between the MWCNT physicochemical properties listed in Table 1 indicated that MWCNT length, diameter and Brunauer–Emmett–Teller (BET) surface area were strongly correlated. Surface area was negatively correlated to diameter and length (Table S5). Therefore, the BET surface area was used to combine the fiber diameter and length into a single parameter. The multiple regression analysis (Table S6A + B) showed that diameter was the best predictor of *IL-8* expression in A549 and THP-1a cells. However, since *IL-8* induction in both A549 and THP-1a cells was predicted by diameter, length and surface area, we were unable to discern between these properties. Surface area was chosen as the proxy for diameter and length, as this parameter allows for assessment of dose-response relationships. When assessing the MWCNT-caused pro-inflammatory response in a co-culture model, statistically significantly increase *IL-8* expression was only detected after exposure to the high dose of NM-401 for 24 h, compared to control cells, as shown in Fig. S2.

#### Genotoxicity

**Fig. 4:**
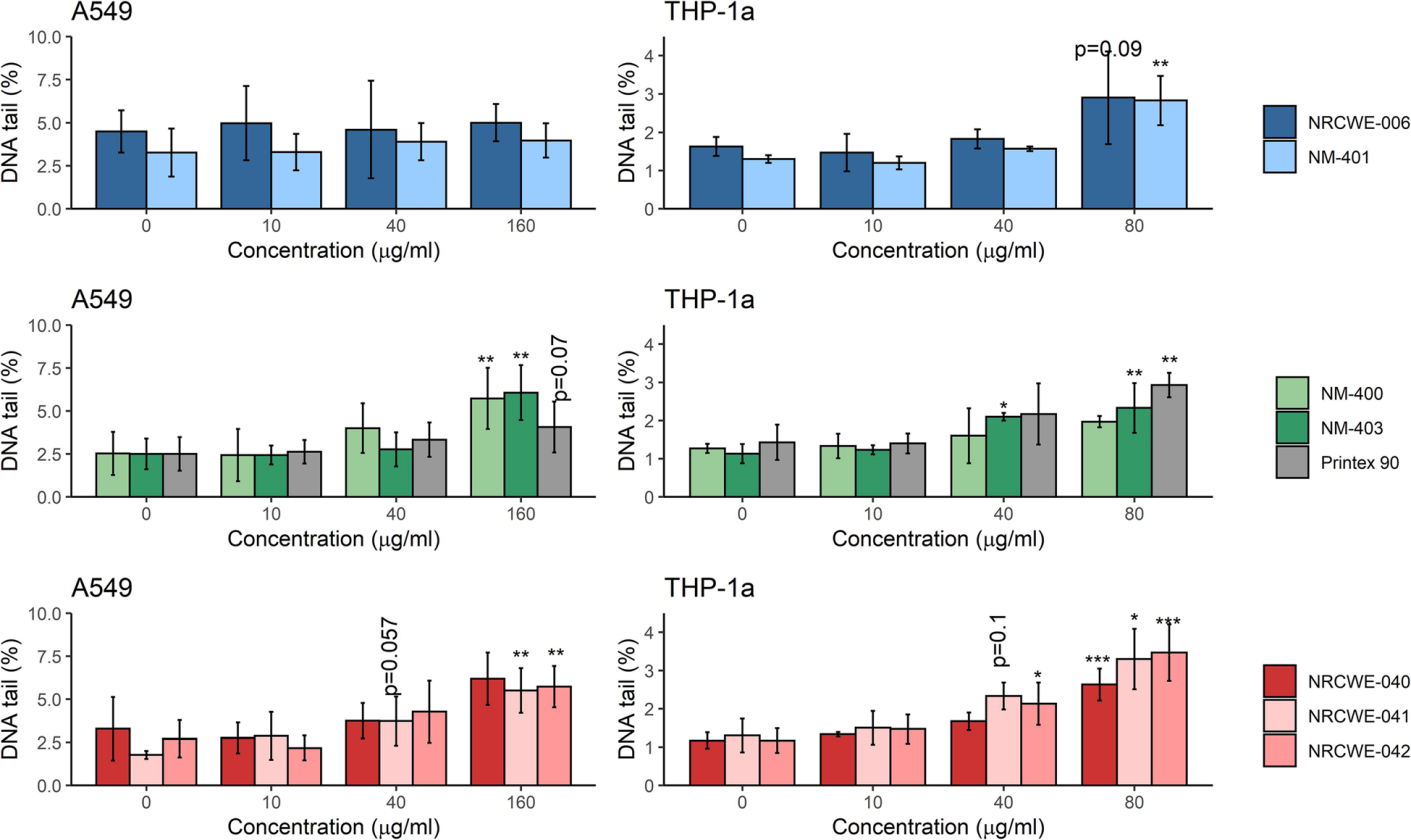
DNA damage in terms of DNA tail (%) in A549 and THP-1a cells exposed to NM for 24 h. The values are mean ± standard deviation of minimum three independent experiments. *: *p*≤0,05, **: *p*≤0.01, ***: *p*≤0.001

We assessed DNA strand breaks levels by alkaline comet assay. A549 and THP-1a cells were exposed as mono-cultures in submerged conditions for 6 or 24 h. The 6 h exposure only increased the level of DNA strand breaks in the A549 and THP-1a cells after exposure to the high concentration of NRCWE-042 (Fig. S3). After 24 h, all NM, except long MWCNT (NRCWE-006 and NM-401) caused a concentration-dependent increase in DNA strand breaks in A549 cells. The high concentration (160 μg/ml) of short MWCNT (NM-400 and NM-403, respectively), as well as surface modified MWCNT (NRCWE-041 and NRCWE-042) increased the level of DNA strand breaks by 2-fold in A549 cells (Fig. 4). Concentration-dependent increase was seen also in A549 cells exposed to CB, although the effects at high concentration (160 μg/ml) only approached statistical significance (*P* = 0.07).

Statistically significant increases in DNA strand break levels were detected at medium and high concentrations (40 and 80 μg/ml, respectively) of NM-403 in THP-1a cells. High concentrations (80 μg/ml) of long MWCNT (NRCWE-006), of pristine (NRCWE-040) and surface-modified (NRCWE-041 and NRCWE-042) MWCNT, and CB, caused statistically significant increases in THP-1a cells. A concentration-dependent increase in DNA strand breaks was seen in THP-1a cells exposed to NM-401, however the effect only approached significance threshold (*p* = 0.09, ANOVA).

No change in DNA strand break levels was detected in the co-culture of A549 and THP-1a cells following ALI exposure to NM-400 or NM-401 (Fig. S2).

The level of DNA strand breaks has been reported as % tail DNA because this is regarded as the most informative primary comet assay descriptor [47]. The presence of NM in the agarose gels is not likely to be a problem because % tail DNA is the fluorescence in the tail relative to the head of comets, whereas NM might interfere with measurements of absolute fluorescence. Measuring DNA migration as tail length is likely not affected by a possible NM interference, therefore we compared tail length to % tail DNA. In our dataset, there is a strong correlation between % tail DNA and tail length (Fig. S4). It should also be noted that we have observed essentially similar results in a separate study on FE1-MutaMouse Lung epithelial cell exposed for MWCNT-7, NM-401 and NM-403 using visual classification of comets [48].

#### In vitro-in vivo correlations

**Fig. 5:**
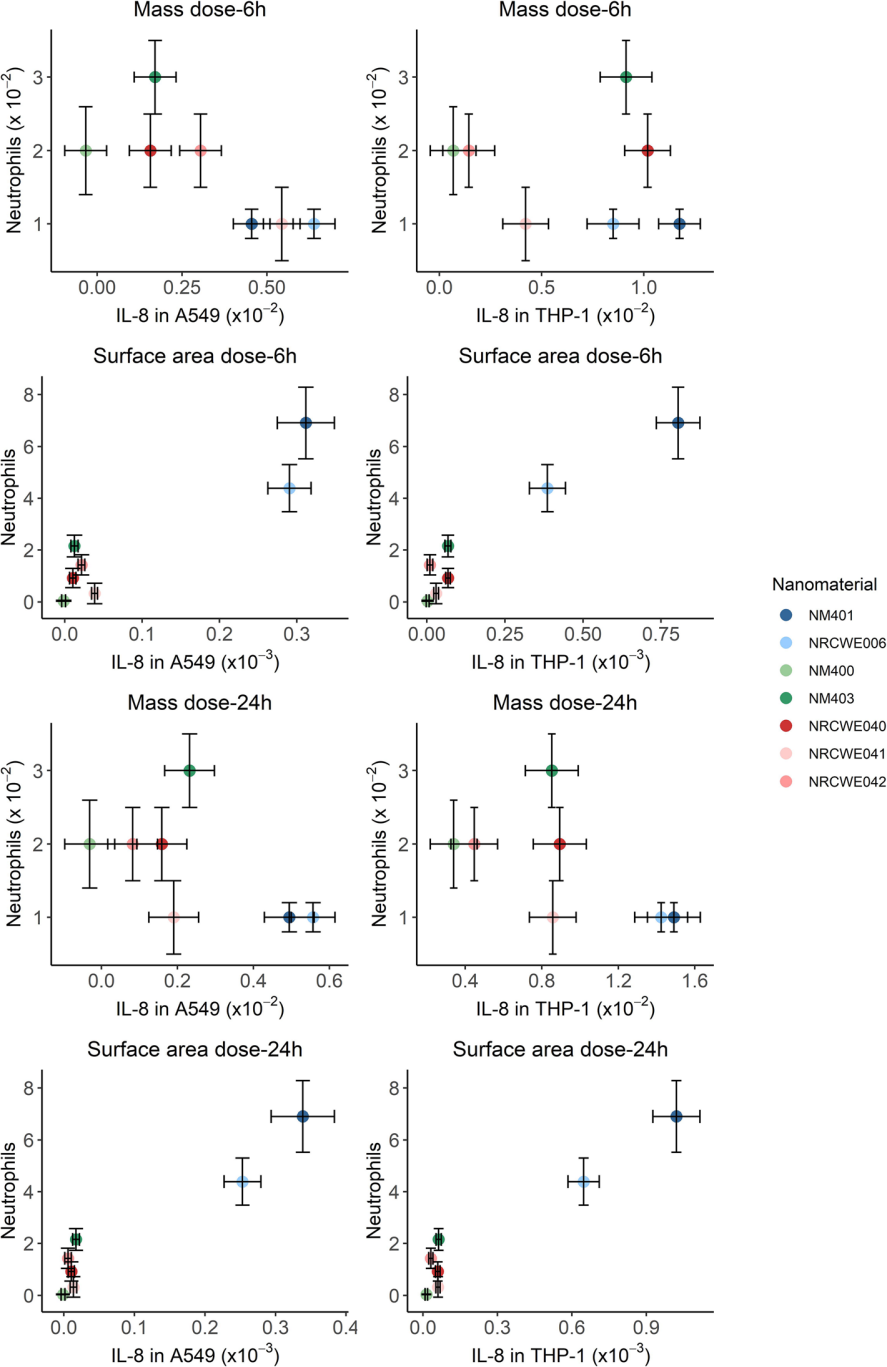
Correlation of the dose-response relationships of the in vivo inflammatory potential in terms of neutrophil influx in mice at day one post-instillation with MWCNT, and the in vitro pro-inflammatory potential in A549 or THP-1a cell mono-cultures exposed in submerged conditions. Dose-response response relationships using mass and surface area are depicted in the top and bottom panels, respectively. The whiskers represent the SEM of the regression estimates based on several doses, and not represented when lower than 0.005

The potential of MWCNT in inducing pro-inflammatory response in vitro, in terms of gene expression levels of *IL-8* in A549 and THP-1a cells, was compared to inflammatory potential in terms of neutrophil influx in BAL fluid in exposed mice [11, 12, 22, 25] (NM-403 in Table S2). When doses were expressed as mass in the regression analysis of the in vivo data, the short MWCNT appeared to be more potent than the long ones, with NM-403 and NRCWE-026 showing the steepest slopes. However, when doses were expressed as surface area (proxy of diameter and length), the regression analysis showed NM-401 to be most potent with highest slope, followed by NRCWE-006 and NM-403, and the other MWCNT in vivo (Fig. S5)*.* Similarly, in vitro, NM-401 and NRCWE-006 appeared more potent than the other MWCNT, with higher slopes in both cell lines, when doses were expressed as surface area (Fig. S5). The slopes of the in vivo inflammatory potential based on neutrophil influx were correlated to the slopes of pro-inflammatory potential in A549 or THP-1a cells, resulting in strong correlation coefficients (*r*) between in vitro and in vivo responses when doses were expressed as surface area (*r =* 0.92 and 0.95 for A549 (6 and 24 h respectively) vs BAL cells, and *r =* 0.96 for THP-1a (both 6 and 24 h) vs BAL cells) (Fig. 5).

**Fig. 6:**
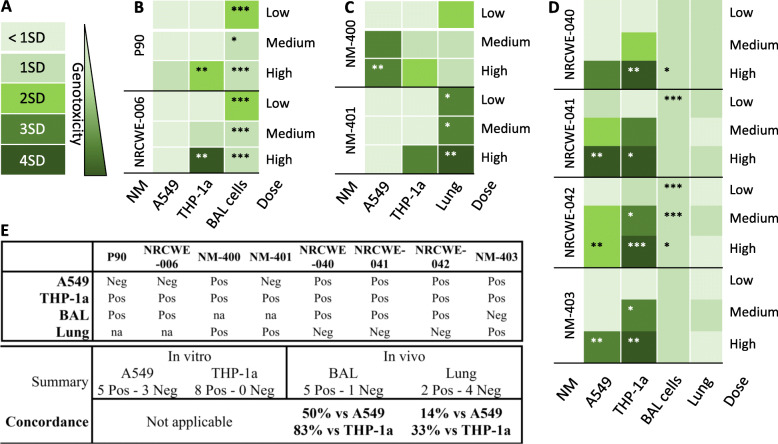
Heat map for in vitro*-*in vivo comparison of genotoxicity potential. Low medium and high dose were different between A549, THP-1a cells and in mice, respectively. The stars represents significantly different DNA damage with respect to control within each NM toxicity test. **A** Heatmap for carbon black (P90) and NRCWE-006, which included BAL cells. **B** Heatmap for NM-400 (NRCWE-026 in vivo) and NM-401, which included lung tissue. **C** Heatmap for NRCWE-040, NRCWE-041, NRCWE-042, and NM-403, which included both BAL cells and lung tissue. **D** Scale of genotoxicity; a material is defined as genotoxic when the effects are ≥2SD higher than their respective control groups. **E** Summary table of correlation results. *, ** and *** designate *P*-values of < 0.05, < 0.01 and < 0.001, respectively in a 2-way ANOVA (see statistics for details)

To compare NM-induced DNA strand breaks by comet assay in vitro and in vivo [11, 22, 25] (NRCWE-006 in Table S7)*,* we used a scoring system based on a categorization of effects into SD-normalized units*.* A NM was classified as genotoxic if the fold increase in DNA strand breaks levels in any dose group was > 2 SD, and/or a statistical significant effect in any dose group (depicted as stars from ANOVA test). The in vitro*-*in vivo comparisons for genotoxicity are shown as heat maps as well as ANOVA test in Fig. 6. All NM were classified as genotoxic in THP-1a cells, as all NM induced significant effects and/or ≥ 2SD difference. In A549 cells, 5 NM were classified as genotoxic, including the surface-modified MWCNT (NRCWE-041 and NRCWE-042), and the short MWCNT (NM-400 and NM-403). In BAL cells, all materials except NM-403 were genotoxic. In lung tissue, NM-401 and NRCWE-006 were genotoxic, with NM-401 showing statistically significantly increased levels of DNA strand breaks in all dose groups and ≥ 3 and 4 SD difference. A 2SD increase was observed at the low dose for NM-400. The concordance is 83% for the binary classification of genotoxicity in THP-1a cells and BAL cells (83%), however poorer concordances are seen for other comparisons (33% for THP-1a and lung tissue; 50% for A549 cells and BAL cells; 14% for A549 and lung tissue).

## Discussion

The aim of this study was to assess how pro-inflammatory responses and genotoxicity caused by different MWCNT in pulmonary cell models correspond to in vivo inflammation and genotoxicity caused by the same materials. Long and thick MWCNT induced strongest pro-inflammatory responses in A549 or THP-1a mono- and co-cultures. All MWCNT induced concentration-dependent increase of DNA strand breaks in THP-1a, whereas only the short MWCNT induced DNA strand breaks in A549 cells. Strong correlation was observed between the pro-inflammatory response in in vitro models in submerged exposure conditions and the in vivo inflammation*,* when pro-inflammatory response and inflammation were normalized to surface area (proxy for diameter and length). We observed a concordance of 83% for genotoxicity in THP-1a and BAL cells. The other in vitro-in vivo combinations resulted in a 14–50% concordance for genotoxicity. In vivo genotoxicity also includes secondary events which may explain some of the differences observed.

To quantify the effective doses of NM to which cells were exposed under submerged condition, we used TGA-MS on cells harvested after 24 h exposure to MWCNT. The importance of NM effective dose for toxicity assessment in vitro has been widely discussed [49]. Notably, when testing under submerged conditions, a particle suspension is administered to the cells adherent to the bottom of a culture well and particle sedimentation occurs over time. When only administered doses are reported, the amount of particles actually reaching cells is unknown and this may hamper the comparison of dose-effect responses, potentially leading to misinterpretations of toxicity [50]. To overcome this, a dosimetry model has been previously developed to predict the effective cell-delivered doses [51], however this has not yet been validated for high-aspect ratio NM like carbon nanotubes [52]. In this study, we employed the TGA-MS to quantify the content of elemental carbon in cells exposed to MWCNT and CB (Table 1). The percentage of administered doses reaching the cells were similar for A549 and THP-1a cells for all studied NMs, except for CB, where effective dose appeared to be larger for THP-1a than A549 cells. This might be explained by the larger agglomerate size of CB when dispersed in RPMI-1640 medium compared to Hams-F12 (Table 1). The TGA-MS method provides a precise quantification of carbon content in cell models exposed in submerged conditions to high aspect ratio MWCNT, and has not been previously explored.

We assessed the pro-inflammatory response in vitro by quantifying expression levels of *IL-8*, a neutrophil chemo-attractant highly expressed during inflammation in both human and murine cells (mouse homologue gene is *Cxcl-1*), as a result of the NF-kβ inflammatory pathway [35, 36]. *IL-8* mRNA levels correlate with IL-8 protein levels in A549 cells [26]. We generally observed all materials to induce a stronger pro-inflammatory response in THP-1a than in A549 cells, likely due to THP-1a phagocytic activity, with the possibility for some long MWCNT also causing frustrated phagocytosis [53]. Of all materials tested, long and thick MWCNT induced strongest pro-inflammatory response in both cell lines. Likewise, a previous study reported high *Cxcl-1* expression in rat macrophages exposed to NM-401, and low or no changes in the cells exposed to NM-403 or NM-400 [54]. The pro-inflammatory response caused by NM-401 was previously linked to the piercing activity of this MWCNT on the cell membranes [8, 54]. Accordingly, long and rigid fibers might have pierced the cell membrane in this study (Fig. S6). In contrast to the present study, NRCWE-006 (Mitsui-7) did not induce *IL-8* expression in A549 cells exposed to MWCNT, when these were dispersed in Pluronic [35]. Different dispersion media have been shown to affect the dispersion state and ultimately the endpoints in vitro [55] and in vivo [56], which might explain some diverging results. Conflicting results are available for inflammation of NM-400; Cao et al., 2016 did not observe secretion of IL-8 protein in A549 cells exposed to 8 μg/ml NM-400, however a significant increase was detected in THP-1a cells [57]. Others observed significantly increased levels of *IL-8* expression with exposure to NM-400 in bronchial epithelial cells (16-HBEC cells) [58]. We observed no pro-inflammatory response in either cell line when exposed to CB, although previous studies have shown significant increase of reactive oxygen species (ROS) production in A549 and THP-1a cells [14, 59]. Hydroxylation and carboxylation of MWCNT (NRCWE-041 and -042) caused similar or slightly increased pro-inflammatory response in A549 (Fig. 2), but a decrease in THP-1a cells (Fig. 3), compared to the pristine (NRCWE-040). Carboxylation of carbon nanotubes has previously been suggested to prevent the permeabilization of phagolysosomes in macrophages, which otherwise could leak proteases into the cytosol and activate a pro-inflammatory response [16]. On the contrary, others have shown that COOH-functionalization of MWCNT increased the activation of NF-kb and oxidative stress in rat macrophages [34, 60]. The effective dose of the studied NMs differed by a factor of ~ 2 between materials, and the ranking of pro-inflammatory potential of NMs in the two cell lines was not affected by whether effective dose or administered dose was used, as elaborated in the Supplemental information (Table S8 and Table S9). The assessment of pro-inflammatory response in co-cultures exposed in the ALI showed that only NM-401 induced significant increase in *IL-8* expression (Fig. S2). In addition, the data indicate that, after administered doses in submerged conditions are adjusted to effective doses (Table 1), NM-401 induced a pro-inflammatory response in the ALI co-culture equal to that induced in A549, but weaker than that induced in THP-1a (Fig. S2).

We quantified DNA strand breaks in cells exposed to MWCNT. DNA damage caused by MWCNT has been linked to ROS production and oxidative stress [61]. We have previously shown large ROS production by DCFH oxidation assay with short MWCNT, including NRCWE-026 (same product but a different batch of NM-400; contains more Al_2_O_3_) [14], NM-403, as well as NRCWE-040, − 041, and − 042 [14, 62]]. This might explain the higher levels of DNA strand breaks in cells exposed to short MWCNT. The lack of DNA damage in epithelial cells (A549) exposed to long MWCNT is consistent with previous observations [14]. We have previously shown that intra-tracheal instillation of mice with doses between 18 and 162 μg of NRCWE-006 and 6 and 54 μg of NM-401 induced statistically significant increases in DNA strand break levels at 1 day post-exposure, in BAL cells and lung tissue, respectively [12, 22]. Similarly, high levels of DNA strand breaks were observed in mouse lung tissue for all doses [22]. Accordingly, length of MWCNT has been suggested as predictor of genotoxicity [62]. The unaltered levels of DNA strand breaks in A549 cells exposed to long MWCNT might indicate the limitation of testing some NM, such as stiff and long MWCNT, in 2D cell cultures; the A549 might be too static to obtain the effects observed in vivo*,* where damage could be generated by the stiff fibers in a moving lung. The limited number of independent replicates included in this study might have compromised the statistical power in those treatments where dose-response relationship in DNA strand breaks is evident (A549 exposed to CB, and THP-1a exposed to NM-400); we have previously reported significantly increased levels of DNA strand breaks in FE1 Muta-Mouse lung epithelial cells (MML) exposed to 100 μg/ml of CB [32], when 5 independent replicates were performed. No DNA damage was detected in cell co-cultures exposed to NM-400 and NM-401 in the ALI system, which is in contrast to what was observed in submerged mono-cultures (Fig. S2).

Length, diameter and surface area were highly correlated in the material data set used in this study, and surface area was used as proxy for diameter and length. When doses were normalized to surface area (as a proxy of diameter and length) rather than mass, we observed strong correlations between the pro-inflammatory potential in vitro, in terms of MWCNT-induced *IL-8* expression in A549 and THP-1a cell mono-cultures, and the in vivo inflammatory potential in terms of MWCNT-induced neutrophil influx in mice lungs [11, 12, 41] (NM-403 data in Table S2). Since length, diameter and surface area were strongly correlated in the in vitro dataset (Table S5 and S6), we are unable to separate the effects of surface area, length and MWCNT diameter on *IL-8* expression in cells. Thus, increased length or diameter predicted increased pro-inflammatory potential. In support of this, MWCNT length and diameter grouped with increasing toxicity in BEAS-2B cells in a recent large study [63]. Jagiello et al. demonstrated that an increasing aspect ratio causes a decrease in the benchmark dose values (increasing effect) of pathways involved in lung inflammation after exposure to MWCNT [64]. As also observed in our study this was seen in particular for NRCWE-006 and NM-401. The correlation of *IL-8* expression with diameter and length indicates that *IL-8* is a good biomarker to assess the pro-inflammatory response caused by high-aspect ratio NM. In addition, the low fold-changes in cells exposed to e.g. CB and NM-400, which were found to be more potent in vivo*,* suggest that end-points involving other inflammatory pathways, might better predict their effects.

While dose-dependent DNA damage could be identified in vitro, some MWCNT result in bell-like-shaped curves when tested in vivo*.* The genotoxicity of NRCWE-006 (also known as Mitsui-7) is supported by evidence of carcinogenicity in rats and mice [65]. NRCWE-006 is biopersistent in the lung and accumulates in the pleural cavity, causing chronic inflammation, fibrosis and cancer [4, 27, 66]. Furthermore, Mitsui-7 is classified as possibly carcinogenic to humans (group 2B) by IARC, based on animal experimental data. The genotoxic potential of long MWCNT is further supported by the present analysis of DNA strand breaks in lung tissue of mice exposed to NM-401 (Fig. 6). For short, thin and entangled MWCNT, the in vivo evidence on carcinogenicity is less clear. One study reported absence of carcinogenicity following intraperitoneal exposure to a high dose of a short and thin MWCNT (length ~ 0.7 μm; diameter: 11.3 ± 3.9 nm) in rats [66], whereas pulmonary exposure to a thin and entangled MWCNT (agglomerate length 1.04 ± 0.71 μm; diameter: 7.4 ± 2.7 nm) caused lung cancer in rats [5].

## Conclusion

In this study, we assessed how MWCNT-induced inflammation and genotoxicity correlate between in vitro and in vivo models. We observed strong correlations between MWCNT-induced pro-inflammatory response in both A549 and THP-1a cells, and in vivo neutrophil influx. For genotoxicity a concordance of 83% was observed for MWCNT-induced DNA damage between THP-1a cells and BAL cells*.* Other in vitro-in vivo combinations resulted in 14–50% concordance.

## Supplementary Information

**Additional file 1 Table S1.** Physicochemical properties of the materials included in this study. **Table S2.** Inflammation and genotoxiciy data from mice exposed to NM-403 via intra-tracheal instillation. **Table S3.** Assignment of genotoxic categories according to SD fold difference. **Table S4.** Viability of A549 and THP-1a after exposure to MWCNT and carbon black for 6 and 24 h (A), and A549 and THP-1a cell co-cultures after exposure for 24 h in the ALI system (B). Control for interference of long or short MWCNT with cell viability measurement (C). **Table S5.** Pearson Correlations of physical chemical properties of MWCNT included in this in vitro study. **Table S6A.** Multiple regression analysis for pro-inflammatory response (IL-8 mRNA levels) at 6 and 24 h in A549 and THP-1a cells. **Table S7.** Genotoxicity data from mice exposed to NRCWE-006 via intra-tracheal instillation. **Table S8.** Ranking of NMs in IL-8 gene induction in A549 for administered doses (160 μg) and effective doses quantified by TGA-MS. **Table S9.** Ranking of NMs in IL-8 gene induction in T for administered doses (160 μg) and effective doses quantified by TGA-MS. **Fig. S1.** Validation of the thermo-gravimetric analysis with quantification of NWCE-006 deposited doses onto the A549 epithelium. Mean of mass exchange (mg) of three independent experiments: 0.48 ± 0.06 (SD). **Fig. S2.** Inflammation (A) quantified as IL-8 gene expression from a co-culture of A549 and THP-1a cells at the apical site. Fibroblasts (WI-38) were cultured on the basolateral side. No significant changes were observed for DNA strand breaks (B) quantified as DNA tail (%) after 24. The doses were quantified with quartz crystal microbalance (C). The data are represented as mean value of 4 independent replicates ± SEM. Star represents statistical significance (*p* < 0.05). **Fig. S3.** DNA damage in terms of % tail DNA in A549 and THP-1a cells exposed to NM for 6 h. The values are mean ± standard deviation of minimum three independent experiments. Statistical significance assessed at *p* ≤ 0.05. **Fig. S4.** Illustration of the linear relationship between %Tail DNA and Tail length in the comet assay. Measuring DNA migration as tail length is likely not affected by a possible NM interference, therefore we compared tail length to % Tail DNA. In our dataset, there is a strong linear correlation THP-1a (*p* < 0.001) and A549 (*p* = 0.005) between % tail DNA and tail length following 24 h of exposure to all tested NM. **Fig. S5.** Regression of dose (in mass and surface area) with inflammation of MWCNT in vitro and in vivo. The slopes were obtained from linear regresions to assess the effect of MWCNT type and surface area on the responses (IL-8 and neutrophil influx). The in vitro data from A549 and THP-1a at 6 and 24 h were combined. NRCWE-026 which was tested in vivo corresponds to NM-400 in vitro; these are the same material, but produced in different batches. **Fig. S6.** Bright field microscopy images of A549 and THP-1a cells exposed to MWCNT and carbon black. Cells were exposed to 40 μg/ml of the materials for 24 h, after which trypsinized, and frozen at − 80 C in freezing media containing FBS and DMSO, until fixation. Cells were stained with Geimsa and scored qualitatively at 100x magnification. The samples shown in the figure were selected as representative of the samples.

## Data Availability

The datasets used and/or analyzed during the current study are available from the corresponding author on a reasonable request.
