# Supplementary material for: In vitro-in vivo correlations of pulmonary inflammogenicity and genotoxicity of MWCNT
Source: Part Fibre Toxicol. 2021 Jul 23;18:25. doi: 10.1186/s12989-021-00413-2 (PMC8299626; doi:10.1186/s12989-021-00413-2)
Supplement: Supplementary file 1 — Additional file 1 Table S1. Physicochemical properties of the materials included in this study. Table S2. Inflammation and genotoxiciy data from mice exposed to NM-403 via intra-tracheal instillation. Table S3. Assignment of genotoxic categories according to SD fold difference. Table S4. Viability of A549 and THP-1a after exposure to MWCNT and carbon black for 6 and 24 h (A), and A549 and THP-1a cell co-cultures after exposure for 24 h in the ALI system (B). Control for interference of long or short MWCNT with cell viability measurement (C). Table S5. Pearson Correlations of physical chemical properties of MWCNT included in this in vitro study. Table S6A. Multiple regression analysis for pro-inflammatory response (IL-8 mRNA levels) at 6 and 24 h in A549 and THP-1a cells. Table S7. Genotoxicity data from mice exposed to NRCWE-006 via intra-tracheal instillation. Table S8. Ranking of NMs in IL-8 gene induction in A549 for administered doses (160 μg) and effective doses quantified by TGA-MS. Table S9. Ranking of NMs in IL-8 gene induction in T for administered doses (160 μg) and effective doses quantified by TGA-MS. Fig. S1. Validation of the thermo-gravimetric analysis with quantification of NWCE-006 deposited doses onto the A549 epithelium. Mean of mass exchange (mg) of three independent experiments: 0.48 ± 0.06 (SD). Fig. S2. Inflammation (A) quantified as IL-8 gene expression from a co-culture of A549 and THP-1a cells at the apical site. Fibroblasts (WI-38) were cultured on the basolateral side. No significant changes were observed for DNA strand breaks (B) quantified as DNA tail (%) after 24. The doses were quantified with quartz crystal microbalance (C). The data are represented as mean value of 4 independent replicates ± SEM. Star represents statistical significance (p < 0.05). Fig. S3. DNA damage in terms of % tail DNA in A549 and THP-1a cells exposed to NM for 6 h. The values are mean ± standard deviation of minimum three independent experiments. Statistical s [file 12989_2021_413_MOESM1_ESM.docx]

**Supplementary information**

**In vitro-in vivo correlations of pulmonary inflammogenicity and genotoxicity of MWCNT**

**Emilio Di Ianni^a^**, Johanna Samulin Erdem^b^, Peter Møller^c^, Nicklas Mønster Salgren^a^, Sarah Søs Poulsen^a^, Kristina Bram Knudsen^a,e^, Shan Zienolddiny^b^, Anne Thoustrup Saber^a^, Håkan Wallin^b^, Ulla Vogel^a,d^, Nicklas Raun Jacobsen^a *^

^a^*National Research Centre for the Working Environment, DK-2100 Copenhagen, Denmark. ^b^National Institute of Occupational Health, Oslo, Norway. ^c^Department of Public Health, University of Copenhagen, Copenhagen, Denmark. ^d^Department of Health Technology, Technical University of Denmark, DK-2800 Kgs.Lyngby, Denmark. ^e^Evaxion Biotech, DK-1260 Copenhagen, Denmark**Correspondence: [nrj@nrcwe.dk](mailto:nrj@nrcwe.dk)

*Assessment of pro-inflammatory response and DNA damage in cell co-cultures*

In order to study the effects of MWCNT in a more complex cell model, we studied the toxicity in co-cultures of A549 and THP-1a cells, which were in contact with lung fibroblasts (WI-38). A549 cells were cultured in RPMI-1640 medium with 10% FBS and 1% P/S. A549 cells were seeded on cell culture inserts (BD Falcon; Porsgrunn, 3696 Norway) with a pore size of 0.4 µm at a density of 3.36x10^5^ cells/ml, and cultured for 5 days in 50:50 RPMI-1640 and DMEM medium. Cell culture medium was changed every other day. THP-1 cells were differentiated into macrophages (THP-1a) in a cell culture flask at a density of 3x10^5^ cells/ml with 10 ng/ml of PMA for 48h. Twenty-four hours prior to exposure, THP-1a cells were detached from the flask with trypsin, centrifuged, and added to the apical side of the insert containing A549 cells, at a ratio of 1:10 of THP-1a and A549 cells, respectively. After ~3 h, the cell culture medium was removed in order to acclimatize the A549 and THP-1a co-culture cells to the ALI. Twenty-four hours prior to exposure, WI-38 fibroblasts were added to the bottom of the well and cultured in 50:50 RPMI-1640 and DMEM medium. The co-culture of A549 and THP-1a cells were in contact with the WI-38 fibroblasts via cell medium.

For testing the effect of MWCNT in an ALI system, we selected NM-400 and NM-401 since these are well characterized materials from the European Joint Research Centre repositories, and represent the short/thin and long/thick groups of MWCNT, respectively. These fibers were prepared according to Jensen et al., 2011[39]. Briefly, ~12 mg of particles were suspended in milliQ-water with 0.05% BSA, to obtain particle suspensions of 2 mg/ml. After sonication, the particles suspensions were filtered through a 40 µm filter and 0.0002 and 0.0004% of NaCl was added to the particle suspension to aid nebulization of NM-400 and NM-401, respectively. A slightly larger quantity of NaCl was needed to efficiently nebulize NM-401. The particle suspensions were aerosolized through a nebulizer with pore size of 10 µm in the Vitrocell Cloud 6 System (Vitrocell, Waldkirch, Germany), and the aerosolized NM were allowed to settle for 5 min. The effective doses in the ALI exposure were quantified by Quartz-Crystal microbalance. A low and high effective dose of the two MWCNT were obtained; NM-400: 2.3±0.4 and 9.42±0.6 µg/cm^2^; NM-401: 3.64±1.4 and 9.59±0.4 µg/cm^2^ (n=4, Figure S2). Dose selection was based on the optimization of the nebulization and dosimetry performed in two independent experiments as recommended in a guideline by the VITROCELL manufacturer. By using optimal conditions we were able to achieve the highest possible ALI exposures. All cell exposures in the ALI system were replicated in four independent experiments. The results of this experiment are shown in Figure S2. A statistically significant increase in *IL-8* expression was detected only in the co-culture (A549+THP-1a cells) exposed to the high dose of NM-401 for 24h, compared to control cells.
No change in DNA strand break levels were detected in the co-culture of A549 and THP-1a cells following ALI exposure to NM-400 or NM-401.
If the effective doses (Table 1) in submerged conditions are taken into account, the pro-inflammatory response in the co-culture in the ALI system observed for NM-401 results to be comparable to the response in A549 cells exposed under submerged conditions. Namely, 74 µg of NM-401 (from administration of 160 µg), corresponding to 21 µg/cm^2^ in the culture well, led to 10 fold increase of *IL-8* in A549. This is comparable to 5.8 fold increase with 9.6 µg/cm^2^ deposited on the quartz-crystal microbalance in the ALI system. However, the response in THP-1a cells in submerged conditions appeared stronger than the co-culture in the ALI system. Specifically, the effective dose of 31 µg (from 80 µg administered), corresponding to 9 µg/cm^2^, led to 42-fold increase of IL-8 in THP-1a cells exposed under submerged conditions.
As shown in Figure 4 and Figure S2, the results of DNA strand break levels are incongruent.

*Identification of physicochemical properties related to toxicity*To identify the physicochemical properties of MWCNT driving their toxicity, multiple regression analyses were conducted on *IL-8* expression and DNA strand breaks in A549 and THP-1a cells. All dependent variables were expressed as fold change relative to controls. The selected MWCNT properties were: BET surface area, diameter, and length, which are reported in Table 1 in the manuscript. The metal impurities are reported in Table S1; Fe content, Mn content, Ni content, Co content, Mg content and OH content.

Initially, a Pearson Correlation analysis was performed to investigate the pairwise associations between the physicochemical parameters of the MWCNT. All parameters were log2 transformed to reach normality. Several parameters were highly correlated in clusters (Table S5); BET surface area, diameter and length (BET was negatively correlated); Fe and Mg content; Co and Mn content; OH and Ni content. Therefore, for each of these clusters/correlations, a proxy variable was chosen, which best explained the variation of the dataset within the cluster. The chosen proxy variables were then included in the further analyses.

The initial multiple regression analyses of the dependency of physicochemical properties on *IL-8* expression and DNA damage in cells 6 and 24 hours after exposure revealed that: 1. The low variance in the levels of DNA strand breaks in these datasets resulted in poor statistical power in the multiple regression analysis. For this reason, we considered the DNA strand breaks dataset not suitable for multiple regression analyses. 2. The MWCNT dataset was too small to extract meaningful results when more than three variables were included.

Based on these observations, we collapsed Mg, Co, Mn and Ni content to a metal parameter by adding their values. A new Pearson Correlation analysis were conducted on the new parameters. This revealed that the metal parameter clustered with the physical parameters cluster (BET surface area, diameter and length). Diameter was identified as the variable best describing the variation of the dataset of the 4 variables in the cluster. Diameter was therefore chosen as the proxy variable of the cluster in the further analyses. For transparency, analyses with BET surface area as proxy for the cluster are also presented. Fe and OH content were independent. However, as dose is included in all analyses, only one additional variable could be included. Fe content was chosen, as OH correlated significantly with BET surface area (Table S2).

For the final multiple regression analyses, the dependency of dose, diameter (BET surface area) and Fe content on *IL-8* expression in cells 6 and 24 hours after exposure was then assessed. All parameters were log2 transformed to reach normality. Statistical significance was determined at the 0.01 level in the multiple regression analyses, since no other correction for mass-significance was performed.

The multiple regression analyses showed that the diameter thickness of the MWCNT, and thereby the shape, were important for their ability to induce expression of *IL-8* in A549 and THP-1a cells 6 and 24 hours after exposure. In contrast, the iron content of the MWCNT were of limited importance (Table S5).

*Analysis of toxicity ranking with administered and effective doses*

We determined whether the ranking in NM-induced pro-inflammatory response would be different when NM administered doses or effective doses were considered. A549 and THP-1a cells were exposed to 160 and 80 µg/ml, respectively, after which the effective doses were quantified by TGA-MS as described in the manuscript methods. The fold induction of IL-8 in the two cell lines was normalized to administered doses and effective doses to determine a ranking (Table S8 and S9). The ranking in potency to induce IL-8 gene expression remained very similar between the two normalizations in both cell lines. Namely, as shown in Table S8, from administered- to (effective)-dose normalized response, the ranking remained the same for NRCWE-006, NM-401, NM-403 and NM-400, while it changed for NRCWE-040 (4 to 5), NRCWE-041 (5 to 7), NRCWE-042 (6 to 4) and CB (7 to 5). In THP-1a cells (Table S9), from administered- to effective-dose normalized response, NRCWE-006 resulted to be most potent, followed by NM-401, NRCWE-040, NM-403, NRCWE-041, NM-400, NRCWE-042 and CB. This comparison was not possible for DNA strand breaks levels, due to the low dynamic range in response and the small (~2fold) variation in effective doses.

**Table S1. Physicochemical properties of the materials included in this study**

| **Nanomaterial** | **Fe_2_O_3_** | **CoO** | **NiO** | **MgO** | **MnO** | **OH**  **mmol/g** |
| --- | --- | --- | --- | --- | --- | --- |
|  | **weight % of the metal oxides** | | | | |  |
| NRCWE-040^1^ | 0.200 | 0.0010 | 0.5600 | 0.010 | 0.002 | 0.35 |
| NRCWE-041^1^ | 0.130 | 0.0010 | 0.3100 | 0.020 | 0.001 | 1.69 |
| NRCWE-42^1^ | 0.080 | 0 | 0.2100 | 0.030 | 0.001 | 4.09 |
| NRCWE-006^2^ | 0.080 | - | - | 0.013 | - | 0.08 |
| NM-400^2^ | 0.260 | 0.1063 | 0.0011 | - | - | 0.79 |
| NM-401^2^ | 0.050 | - |  | 0.015 | - | 0.03 |
| NM-403^2^ | 0.002 | 1.2000 | 0.0018 | 0.188 | 0.160 | 0.19 |
| Printex 90^3^ | - | - | - | - | - | - |

The nanomaterials were previously characterized. Data are extracted from ^1^ Poulsen et al. 2016, ^2^ Jackson et al., 2015 and ^3^ Jacobsen et al., 2008.

– Not detected.

**Table S2. Inflammation and genotoxiciy data from mice exposed to NM-403 via intra-tracheal instillation.**

| Dose instilled | Neutrophil influx in BAL fluid (*10^3^) | DNA tail (%) in BAL cells | DNA tail (%) in lung tissue |
| --- | --- | --- | --- |
| Vehicle | 5.0 ± 0.8 | 2.9 ± 1.0 | 3.1 ± 2.1 |
| 6 µg | 27.1 ± 4.6* | 4.5 ± 1.1 | 2.7 ± 0.4 |
| 18 µg | 144.9 ± 24.0*** | 3.8 ± 0.5 | 3.4 ± 0.4 |
| 54 µg | 150.2 ± 21.9*** | 4.3 ± 0.8 | 3.0 ± 0.5 |

Mice were exposed for one day. Inflammation is reported as mean ± SEM. DNA damage as mean ± SD. The stars represent significance at p<0.05 (one-way-ANOVA).

**Table S3. Assignment of genotoxic categories according to SD fold difference.**

|  | **A549 cells** | **THP-1a cells** | **BAL cells** | **Lung tissue** |
| --- | --- | --- | --- | --- |
| **Category** | **Fold-difference** | **Fold-difference** | **Fold-difference** | **Fold-difference** |
| **<1 SD** | <1.43 | <1.22 | <1.00 | <1.00 |
| **1 SD** | >1.43 | >1.22 | >1.00 | >1.00 |
| **2 SD** | >1.87 | >1.45 | >1.78 | >1.53 |
| **3 SD** | >2.31 | >1.67 | >2.56 | >2.07 |
| **4 SD** | >2.74 | >1.90 | >3.34 | >2.61 |

Mean levels of DNA strand breaks were 2.9 ± 1.26 (A549 cells), 1.3 ± 0.29 (THP-1a cells), 7.37 ± 5.76 (BAL cells), 3.79 ± 2.02 (lung tissue), respectively. Categorization of inflammatory and genotoxic effect in cell cultures and animals (heat map). The heat map represent the difference in effect size between exposed and controls in terms of SD units. Table S1 shows the relationship between color indication in the heat map and SD units. In reference, the table outlines the effect size in fold-difference, using this equation below:

$$Fold difference=\frac{Mean \% tail DNA + (\left( n \right)*SD))}{Mean \% tail DNA}$$

Mean % tail DNA and standard deviation (SD) were calculated for the pooled control group.

**Table S4. Viability of A549 and THP-1a after exposure to MWCNT and carbon black for 6 and 24h (A), and A549 and THP-1a cell co-cultures after exposure for 24h in the ALI system (B). Control for interference of long or short MWCNT with cell viability measurement (C).**

|  |  | |  |  | | |  | | | |  | |  |  |  |  |  |  |  |
| --- | --- | --- | --- | --- | --- | --- | --- | --- | --- | --- | --- | --- | --- | --- | --- | --- | --- | --- | --- |
| **A:** | **A549** | | | | | | | **THP-1a** | | | | | |  |  |  |  |  |  |
| **NM** | **Time (h)** | **Dose  (µg/ml)** | **Viability ± SD  (%)** | | | **Cells ± SD**  **(number * 10^5^)** | | **Time  (h)** | **Dose  (µg/ml)** | **Viability ± SD  (%)** | | **Cells ± SD**  **(number * 10^5^)** | |  |  |  |  |  |  |
| NM-401 | 6 | 0 | 99.5 ± 0.1 | | 11.0 ± 1.1 | | | 6 | 0 | 98.0 ± 0.8 | | 10.5 ± 0.6 | |  |  |  |  |  |  |
| NM-401 | 6 | 10 | 99.3 ± 0.2 | | 10.0 ± 0.0 | | | 6 | 10 | 97.5 ± 0.5 | | 10.2 ± 1.4 | |  |  |  |  |  |  |
| NM-401 | 6 | 40 | 99.3 ± 0.4 | | 10.0 ± 1.6 | | | 6 | 40 | 96.4 ± 0.3 | | 10.1 ± 1.0 | |  |  |  |  |  |  |
| NM-401 | 6 | 160 | 99.1 ± 0.2 | | 11.0 ± 0.3 | | | 6 | 80 | 94.2 ± 2.0 | | 8.33 ± 3.7 | |  |  |  |  |  |  |
| NM-401 | 24 | 0 | 99.5 ± 0.4 | | 16.0 ± 2.1 | | | 24 | 0 | 96.0 ± 0.9 | | 8.31 ± 2.2 | |  |  |  |  |  |  |
| NM-401 | 24 | 10 | 99.3 ± 0.4 | | 17.0 ± 2.6 | | | 24 | 10 | 96.0 ± 2.1 | | 9.90 ± 0.7 | |  |  |  |  |  |  |
| NM-401 | 24 | 40 | 98.6 ± 0.9 | | 17.0 ± 2.8 | | | 24 | 40 | 95.5 ± 2.9 | | 9.39 ± 0.3 | |  |  |  |  |  |  |
| NM-401 | 24 | 160 | 90.9 ± 5.4 | | 11.0 ± 2.4 | | | 24 | 80 | 83.0 ± 6.6 | | 6.10 ± 1.7 | |  |  |  |  |  |  |
| NRCWE-006-00000006 | 6 | 0 | 99.7 ± 0.2 | | 9.7 ± 0.8 | | | 6 | 0 | 94.3 ± 6.6 | | 10.6 ± 1.2 | |  |  |  |  |  |  |
| NRCWE-006 | 6 | 10 | 99.7 ± 0.2 | | 10.0 ± 1.0 | | | 6 | 10 | 94.3 ± 6.3 | | 9.91 ± 0.6 | |  |  |  |  |  |  |
| NRCWE-006 | 6 | 40 | 99.3 ± 0.2 | | 9.1 ± 0.6 | | | 6 | 40 | 94.0 ± 5.1 | | 9.62 ± 0.5 | |  |  |  |  |  |  |
| NRCWE-006 | 6 | 160 | 98.2 ± 0.8 | | 8.2 ± 1.7 | | | 6 | 80 | 92.6 ± 3.8 | | 8.50 ± 1.6 | |  |  |  |  |  |  |
| NRCWE-006 | 24 | 0 | 99.5 ± 0.3 | | 12.0 ± 2.8 | | | 24 | 0 | 96.6 ± 0.6 | | 10.2 ± 1.2 | |  |  |  |  |  |  |
| NRCWE-006 | 24 | 10 | 99.1 ± 0.4 | | 13.0 ± 1.6 | | | 24 | 10 | 97.0 ± 0.2 | | 9.86 ± 0.6 | |  |  |  |  |  |  |
| NRCWE-006 | 24 | 40 | 96.7 ± 1.1 | | 11.0 ± 0.7 | | | 24 | 40 | 95.6 ± 1.1 | | 8.78 ± 1.0 | |  |  |  |  |  |  |
| NRCWE-006 | 24 | 160 | 88.1 ± 4.5 | | 6.8 ± 0.5 | | | 24 | 80 | 75.8 ± 13.1 | | 6.55 ± 1.2 | |  |  |  |  |  |  |
| NM-400 | 6 | 0 | 99.4 ± 0.1 | | 12.0 ± 0.7 | | | 6 | 0 | 88.6 ± 16.3 | | 7.29 ± 2.0 | |  |  |  |  |  |  |
| NM-400 | 6 | 10 | 99.4 ± 0.3 | | 9.7 ± 1.2 | | | 6 | 10 | 94.3 ± 7.3 | | 10.3 ± 2.0 | |  |  |  |  |  |  |
| NM-400 | 6 | 40 | 99.5 ± 0.2 | | 11.0 ± 1.4 | | | 6 | 40 | 91.8 ± 10.7 | | 10.7 ± 1.6 | |  |  |  |  |  |  |
| NM-400 | 6 | 160 | 99.6 ± 0.5 | | 6.9 ± 0.5 | | | 6 | 80 | 92.3 ± 10.0 | | 9.0 ± 2.3 | |  |  |  |  |  |  |
| NM-400 | 24 | 0 | 99.6 ± 0.2 | | 16.0 ± 0.2 | | | 24 | 0 | 97.4 ± 0.9 | | 8.9 ± 1.2 | |  |  |  |  |  |  |
| NM-400 | 24 | 10 | 99.7 ± 0.3 | | 15.0 ± 2.8 | | | 24 | 10 | 97.1 ± 0.5 | | 9.6 ± 0.4 | |  |  |  |  |  |  |
| NM-400 | 24 | 40 | 99.6 ± 0.3 | | 15.0 ± 1.8 | | | 24 | 40 | 96.8 ± 0.7 | | 8.6 ± 0.5 | |  |  |  |  |  |  |
| NM-400 | 24 | 160 | 99.1 ± 0.3 | | 6.2 ± 0.7 | | | 24 | 80 | 95.4 ± 1.1 | | 7.5 ± 0.7 | |  |  |  |  |  |  |
| NM-403 | 6 | 0 | 99.7 ± 0.2 | | 13.0 ± 1.9 | | | 6 | 0 | 96.7 ± 1.4 | | 10.8 ± 0.4 | |  |  |  |  |  |  |
| NM-403 | 6 | 10 | 99.2 ± 0.4 | | 13.0 ± 1.8 | | | 6 | 10 | 96.8 ± 0.8 | | 10.8 ± 0.7 | |  |  |  |  |  |  |
| NM-403 | 6 | 40 | 99.3 ± 0.1 | | 13.0 ± 1.8 | | | 6 | 40 | 96.4 ± 0.4 | | 10.8 ± 0.7 | |  |  |  |  |  |  |
| NM-403 | 6 | 160 | 98.9 ± 0.3 | | 7.8 ± 0.8 | | | 6 | 80 | 90.8 ± 3.4 | | 9.3 ± 1.1 | |  |  |  |  |  |  |
| NM-403 | 24 | 0 | 99.5 ± 0.3 | | 20.0 ± 1.8 | | | 24 | 0 | 96.1 ± 2.5 | | 9.4 ± 1.5 | |  |  |  |  |  |  |
| NM-403 | 24 | 10 | 99.5 ± 0.1 | | 20.0 ± 1.1 | | | 24 | 10 | 94.0 ± 3.6 | | 9.9 ± 1.3 | |  |  |  |  |  |  |
| NM-403 | 24 | 40 | 99.3 ± 0.5 | | 17.0 ± 0.9 | | | 24 | 40 | 94.1 ± 3.9 | | 7.6 ± 0.6 | |  |  |  |  |  |  |
| NM-403 | 24 | 160 | 97.7 ± 0.3 | | 7.5 ± 0.6 | | | 24 | 80 | 86.0 ± 7.8 | | 6.0 ± 1.4 | |  |  |  |  |  |  |
| NRCWE-040 | 6 | 0 | 99.6 ± 0.4 | | 13.0 ± 1.8 | | | 6 | 0 | 97.3 ± 0.8 | | 9.7 ± 2.6 | |  |  |  |  |  |  |
| NRCWE-040 | 6 | 10 | 99.4 ± 0.0 | | 13.0 ± 0.9 | | | 6 | 10 | 97.7 ± 0.9 | | 11.2 ± 0.3 | |  |  |  |  |  |  |
| NRCWE-040 | 6 | 40 | 99.6 ± 0.2 | | 13.1 ± 1.0 | | | 6 | 40 | 97.3 ± 2.0 | | 11.3 ± 0.8 | |  |  |  |  |  |  |
| NRCWE-040 | 6 | 160 | 99.2 ± 0.4 | | 7.8 ± 2.1 | | | 6 | 80 | 93.6 ± 4.3 | | 9.4 ± 1.6 | |  |  |  |  |  |  |
| NRCWE-040 | 24 | 0 | 99.3 ± 0.3 | | 18.0 ± 1.6 | | | 24 | 0 | 97.2 ± 1.4 | | 8.0 ± 3.2 | |  |  |  |  |  |  |
| NRCWE-040 | 24 | 10 | 99.5 ± 0.1 | | 18.0 ± 2.4 | | | 24 | 10 | 97.3 ± 0.9 | | 9.8 ± 0.5 | |  |  |  |  |  |  |
| NRCWE-040 | 24 | 40 | 99.0 ± 0.2 | | 17.0 ± 1.3 | | | 24 | 40 | 94.7 ± 1.0 | | 9.0 ± 0.6 | |  |  |  |  |  |  |
| NRCWE-040 | 24 | 160 | 97.7 ± 1.1 | | 8.6 ± 2.1 | | | 24 | 80 | 90.0 ± 2.2 | | 6.9 ± 1.9 | |  |  |  |  |  |  |
| NRCWE-041 | 6 | 0 | 99.5 ± 0.1 | | 12.0 ± 2.5 | | | 6 | 0 | 91.9 ± 12.1 | | 6.7 ± 3.0 | |  |  |  |  |  |  |
| NRCWE-041 | 6 | 10 | 99.6 ± 0.1 | | 13.0 ± 0.7 | | | 6 | 10 | 98.5 ± 0.4 | | 11.0 ± 1.0 | |  |  |  |  |  |  |
| NRCWE-041 | 6 | 40 | 99.2 ± 0.3 | | 13.0 ± 1.0 | | | 6 | 40 | 98.2 ± 0.8 | | 11.0 ± 0.6 | |  |  |  |  |  |  |
| NRCWE-041 | 6 | 160 | 98.5 ± 0.5 | | 6.8 ± 1.3 | | | 6 | 80 | 95.7 ± 1.9 | | 8.2 ± 1.9 | |  |  |  |  |  |  |
| NRCWE-041 | 24 | 0 | 99.7 ± 0.2 | | 18.0 ± 2.5 | | | 24 | 0 | 98.1 ± 0.3 | | 8.2 ± 1.3 | |  |  |  |  |  |  |
| NRCWE-041 | 24 | 10 | 99.4 ± 0.4 | | 17.0 ± 1.4 | | | 24 | 10 | 96.6 ± 0.3 | | 8.9 ± 0.6 | |  |  |  |  |  |  |
| NRCWE-041 | 24 | 40 | 98.9 ± 0.1 | | 16.0 ± 2.2 | | | 24 | 40 | 95.7 ± 1.1 | | 8.5 ± 0.9 | |  |  |  |  |  |  |
| NRCWE-041 | 24 | 160 | 95.4 ± 1.4 | | 6.6 ± 1.3 | | | 24 | 80 | 91.8 ± 3.7 | | 5.4 ± 2.2 | |  |  |  |  |  |  |
| NRCWE-042 | 6 | 0 | 99.2 ± 0.3 | | 13.0 ± 1.1 | | | 6 | 0 | 97.1 ± 0.3 | | 10.8 ± 1.7 | |  |  |  |  |  |  |
| NRCWE-042 | 6 | 10 | 99.4 ± 0.3 | | 13.0 ± 0.9 | | | 6 | 10 | 97.1 ± 1.2 | | 11.3 ± 0.5 | |  |  |  |  |  |  |
| NRCWE-042 | 6 | 40 | 99.3 ± 0.3 | | 13.0 ± 0.8 | | | 6 | 40 | 96.4 ± 1.6 | | 10.2 ± 0.3 | |  |  |  |  |  |  |
| NRCWE-042 | 6 | 160 | 98.7 ± 1.0 | | 7.6 ± 0.5 | | | 6 | 80 | 95.5 ± 3.9 | | 9.3 ± 0.2 | |  |  |  |  |  |  |
| NRCWE-042 | 24 | 0 | 99.7 ± 0.2 | | 17.0 ± 1.5 | | | 24 | 0 | 95.7 ± 1.8 | | 7.8 ± 2.3 | |  |  |  |  |  |  |
| NRCWE-042 | 24 | 10 | 99.4 ± 0.1 | | 18.0 ± 1.9 | | | 24 | 10 | 96.6 ± 0.3 | | 9.0 ± 0.5 | |  |  |  |  |  |  |
| NRCWE-042 | 24 | 40 | 98.9 ± 0.3 | | 16.0 ± 1.7 | | | 24 | 40 | 94.5 ± 2.6 | | 8.8 ± 0.8 | |  |  |  |  |  |  |
| NRCWE-042 | 24 | 160 | 96.2 ± 1.0 | | 7.2 ± 1.0 | | | 24 | 80 | 86.4 ± 1.9 | | 5.7 ± 1.5 | |  |  |  |  |  |  |
| Printex 90 | 6 | 0 | 99.3 ± 0.2 | | 11.0 ± 1.6 | | | 6 | 0 | 93.8 ± 8.4 | | 10.0 ± 1.5 | |  |  |  |  |  |  |
| Printex 90 | 6 | 10 | 99.5 ± 0.1 | | 12.0 ± 1.1 | | | 6 | 10 | 95.9 ± 4.4 | | 9.1 ± 1.1 | |  |  |  |  |  |  |
| Printex 90 | 6 | 40 | 99.3 ± 0.4 | | 11.0 ± 0.9 | | | 6 | 40 | 97.2 ± 1.8 | | 8.7 ± 1.1 | |  |  |  |  |  |  |
| Printex 90 | 6 | 160 | 99.4 ± 0.3 | | 11.0 ± 0.5 | | | 6 | 80 | 97.7 ± 1.2 | | 6.3 ± 1.6 | |  |  |  |  |  |  |
| Printex 90 | 24 | 0 | 99.6 ± 0.3 | | 16.0 ± 2.2 | | | 24 | 0 | 98.3 ± 0.8 | | 6.9 ± 1.6 | |  |  |  |  |  |  |
| Printex 90 | 24 | 10 | 99.4 ± 0.5 | | 15.0 ± 1.1 | | | 24 | 10 | 98.1 ± 0.7 | | 10.0 ± 0.8 | |  |  |  |  |  |  |
| Printex 90 | 24 | 40 | 99.6 ± 0.2 | | 16.0 ± 0.6 | | | 24 | 40 | 95.7 ± 1.3 | | 7.9 ± 2.1 | |  |  |  |  |  |  |
| Printex 90 | 24 | 160 | 99.3 ± 0.2 | | 14.0 ± 2.0 | | | 24 | 80 | 95.9 ± 0.6 | | 5.1 ± 2.3 | |  |  |  |  |  |  |

| **B: A549-THP-1a Co-cultures** | | | | | | |  |  |  |  |  |  |  |
| --- | --- | --- | --- | --- | --- | --- | --- | --- | --- | --- | --- | --- | --- |
| **NM** | **Time (h)** | | **Dose  (µg/ml)** | | **Viability ± SD  (%)** | |  |  |  |  |  |  |  |
| Control | 24 | | 0 | | 99.7 ± 0.2 | |  |  |  |  |  |  |  |
| NM-400 | 24 | | 2.3 ± 0.4 | | 99.6 ± 0.2 | |  |  |  |  |  |  |  |
| NM-400 | 24 | | 9.4 ± 0.6 | | 99.5 ± 0.4 | |  |  |  |  |  |  |  |
| NM-401 | 24 | | 3.6 ± 1.4 | | 99.5 ± 0.2 | |  |  |  |  |  |  |  |
| NM-401 | 24 | | 9.6 ± 0.4 | | 99.5 ± 0.7 | |  |  |  |  |  |  |  |
|  | |  | | | | | | |  |  | | | |
| **C:** | | **NM-401** | | | | | | |  | **NM-403** | | | |
| **MWCNT conc.** | | **Viability**  **(%)** | | **Viable**  **Cells/ml** | | **Nonviable**  **Cells/ml** | | **Total**  **Cells/ml** |  | **Viability**  **(%)** | **Viable**  **Cells/ml** | **Nonviable**  **Cells/ml** | **Total**  **Cells/ml** |
| 0µg/ml | | 95,1 | | 644000 | | 33600 | | 677500 |  | 94,8 | 726500 | 39750 | 766000 |
| 20µg/ml | | 94,3 | | 609000 | | 36650 | | 645500 |  | 91,8 | 699500 | 62700 | 762500 |
| 40µg/ml | | 94,3 | | 643500 | | 39050 | | 682500 |  | 93,4 | 747000 | 53100 | 800000 |
| 80µg/ml | | 94,2 | | 651500 | | 40450 | | 692000 |  | 90,6 | 504500 | 52100 | 556500 |
| 160µg/ml | | 94,4 | | 634000 | | 38000 | | 672000 |  | 91,8 | 172000 | 15267 | 187000 |
| 320µg/ml | | 92,7 | | 592500 | | 46600 | | 638500 |  | 93,4 | 39050 | 2740 | 41800 |

**Table S5. Pearson Correlations of physical chemical properties of MWCNT included in this in**

**vitro study.**

| **BET** | 1.00 |  |  |  |  |  |  |  |
| --- | --- | --- | --- | --- | --- | --- | --- | --- |
| **Diameter** | **-0.89**** | 1.00 |  |  |  |  |  |  |
| **Length** | **-0.89**** | **0.87*** | 1.00 |  |  |  |  |  |
| **Fe_2_O_3_** | 0.12 | 0.18 | 0.19 | 1.00 |  |  |  |  |
| **MnO** | 0.55 | -0.70 | **-0.78*** | -0.72 | 1.00 |  |  |  |
| **NiO** | 0.66 | -0.33 | -0.68 | 0.29 | 0.30 | 1.00 |  |  |
| **CoO** | 0.58 | **-0.87*** | -0.62 | -0.54 | **0.76*** | -0.11 | 1.00 |  |
| **MgO** | -0.06 | -0.07 | -0.27 | **-0.89**** | 0.72 | 0.07 | 0.28 | 1.00 |
| **OH** | **0.80*** | -0.48 | -0.63 | 0.31 | 0.22 | **0.77*** | 0.10 | -0.05 |
|  |  |  |  |  |  |  |  |  |
|  | **BET** | **Diameter** | **Length** | **Fe_2_O_3_** | **MnO** | **NiO** | **CoO** | **MgO** |

The physico-chemical properties were taken from Table 1 and Table S1. Values in bold represent statistically significant strong correlations at 5 and 1% level, respectively (* or **).

**Table S6A. Multiple regression analysis for pro-inflammatory response (*IL-8* mRNA levels*)* at 6 and 24h in A549 and THP-1a cells.**

| **A549** | | | | **THP-1** | | | |
| --- | --- | --- | --- | --- | --- | --- | --- |
| **Hour** | **Variable** | **Multiplicative effect** | **p value** | **Hour** | **Variable** | **Multiplicative effect** | **p value** |
| **6** | **Per doubling in Dose** | **1.27027** | **<.0001** | **6** | **Per doubling in Dose** | **1.25082** | **<.0001** |
| **6** | **Per doubling in Diameter** | **1.35537** | **<.0001** | **6** | **Per doubling in Diameter** | **1.32295** | **0.0039** |
| 6 | Per doubling in Fe | 0.97611 | 0.4814 | **6** | **Per doubling in Fe** | **0.87315** | **0.0036** |
| **24** | **Per doubling in Dose** | **1.21690** | **<.0001** | **24** | **Per doubling in Dose** | **1.34615** | **<.0001** |
| **24** | **Per doubling in Diameter** | **1.48625** | **<.0001** | **24** | **Per doubling in Diameter** | **1.32430** | **0.0018** |
| 24 | Per doubling in Fe | 0.92258 | 0.0162 | 24 | Per doubling in Fe | 0.93430 | 0.1167 |

Values in bold represent statistically significant regression between *IL-8* expression and included physicochemical properties of the MWCNT. Diameter was chosen as proxy for the cluster, which included BET surface area, length, diameter and metal content.

**Table S6B. Multiple regression analysis for pro-inflammatory response (*IL-8* mRNA levels*)* at 6 and 24h in A549 and THP-1a cells.**

| **A549** | | | | **THP-1** | | | |
| --- | --- | --- | --- | --- | --- | --- | --- |
| **Hour** | **Variable** | **Multiplicative effect** | **p value** | **Hour** | **Variable** | **Multiplicative effect** | **p value** |
| **6** | **Per doubling in Dose** | **1.27027** | **<.0001** | **6** | **Per doubling in Dose** | **1.24943** | **<.0001** |
| **6** | **Per doubling in BET surface area** | **0.86742** | **0.0105** | **6** | **Per doubling in BET surface area** | **0.80030** | **0.0014** |
| 6 | Per doubling in Fe | 1.01279 | 0.7249 | 6 | Per doubling in Fe | 0.90965 | 0.0382 |
| **24** | **Per doubling in Dose** | **1.21690** | **<.0001** | **24** | **Per doubling in Dose** | **1.34741** | **<.0001** |
| **24** | **Per doubling in BET surface area** | **0.74423** | **<.0001** | **24** | **Per doubling in BET surface area** | **0.97143** | **0.0014** |
| 24 | Per doubling in Fe | 0.97588 | 0.4466 | 24 | Per doubling in Fe | 0.81254 | 0.5052 |

Values in bold represent statistically significant regression between *IL-8* expression and included physicochemical properties of the MWCNT. BET surface area was chosen as proxy for the cluster, which included BET surface area, length, diameter and metal content.

**Table S7. Genotoxicity data from mice exposed to NRCWE-006 via intra-tracheal instillation.**

| Dose instilled | DNA tail (%) in BAL cells |
| --- | --- |
| Vehicle | 14.3 ± 5.0 |
| 18 µg | 27.2 ± 3.9 *** |
| 54 µg | 24.8 ± 4.4 *** |
| 162 µg | 22.9 ± 1.9 *** |

Mice were exposed for one day. DNA damage as mean ± SD. The stars represent significance at p<0.05 (one-way-ANOVA).

**Table S8. Ranking of NMs in IL-8 gene induction in A549 for administered doses (160µg) and effective doses quantified by TGA-MS.**

| \| **A549** \| \| \| \| \| \| \| \| \| --- \| --- \| --- \| --- \| --- \| --- \| --- \| --- \| \| **NM** \| **Fold IL8** \| **Administered dose (µg)** \| **Fold IL-8/ µg administered** \| **Ranking** \| **Effective dose (µg)** \| **Fold IL-8/ µg effective** \| **Ranking** \| \| NRCWE-006 \| 18.00 \| 160 \| 0.1125 \| 1 \| 40 \| 0.450 \| 1 \| \| NM-401 \| 9.84 \| 160 \| 0.0615 \| 2 \| 74 \| 0.133 \| 2 \| \| NM-403 \| 4.36 \| 160 \| 0.02725 \| 3 \| 54 \| 0.081 \| 3 \| \| NRCWE-040 \| 3.39 \| 160 \| 0.0211875 \| 4 \| 74 \| 0.046 \| 5 \| \| NRCWE-041 \| 3.12 \| 160 \| 0.0195 \| 5 \| 85 \| 0.037 \| 7 \| \| NRCWE-042 \| 2.35 \| 160 \| 0.0146875 \| 6 \| 40 \| 0.059 \| 4 \| \| CB \| 1.82 \| 160 \| 0.011375 \| 7 \| 40 \| 0.046 \| 5 \| \| NM-400 \| 0.71 \| 160 \| 0.0044375 \| 8 \| 42 \| 0.017 \| 8 \| |
| --- | --- | --- | --- | --- | --- | --- | --- | --- | --- | --- | --- | --- | --- | --- | --- | --- | --- | --- | --- | --- | --- | --- | --- | --- | --- | --- | --- | --- | --- | --- | --- | --- | --- | --- | --- | --- | --- | --- | --- | --- | --- | --- | --- | --- | --- | --- | --- | --- | --- | --- | --- | --- | --- | --- | --- | --- | --- | --- | --- | --- | --- | --- | --- | --- | --- | --- | --- | --- | --- | --- | --- | --- | --- | --- | --- | --- | --- | --- | --- | --- |

**Table S9. Ranking of NMs in IL-8 gene induction in T for administered doses (160µg) and effective doses quantified by TGA-MS.**

| **THP-1** | | | | | | | |
| --- | --- | --- | --- | --- | --- | --- | --- |
| **NM** | **Fold IL8** | **Administered dose (µg)** | **Fold IL-8/ µg administered** | **Ranking** | **Effective dose (µg)** | **Fold IL-8/ µg effective** | **Ranking** |
| NM-401 | 42.96 | 80 | 0.537 | 1 | 31 | 1.386 | 2 |
| NRCWE-006 | 28.10 | 80 | 0.35125 | 2 | 17 | 1.653 | 1 |
| NRCWE-040 | 12.97 | 80 | 0.162125 | 3 | 42 | 0.309 | 3 |
| NRCWE-041 | 8.57 | 80 | 0.107125 | 4 | 46 | 0.186 | 5 |
| NM-403 | 8.36 | 80 | 0.1045 | 5 | 36 | 0.232 | 4 |
| NRCWE-042 | 3.55 | 80 | 0.044375 | 6 | 30 | 0.118 | 7 |
| NM-400 | 2.93 | 80 | 0.036625 | 7 | 29 | 0.101 | 6 |
| CB | 0.77 | 80 | 0.009625 | 8 | 38 | 0.020 | 8 |


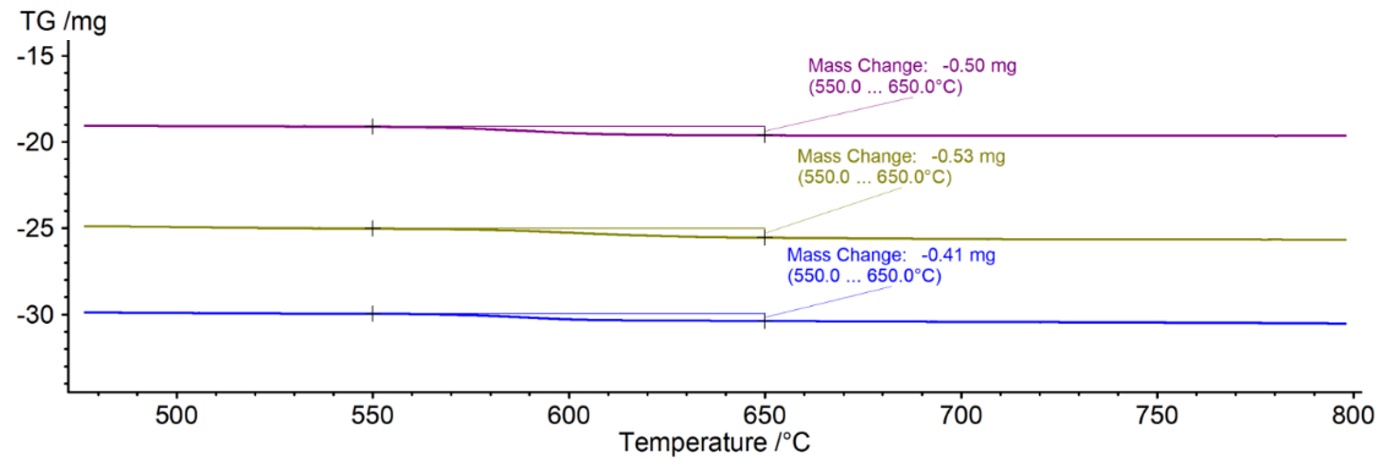

**Figure S1.** Validation of the thermo-gravimetric analysis with quantification of NWCE-006 deposited doses onto the A549 epithelium. Mean of mass exchange (mg) of three independent experiments: 0.48 ± 0.06 (SD).

| **A** | 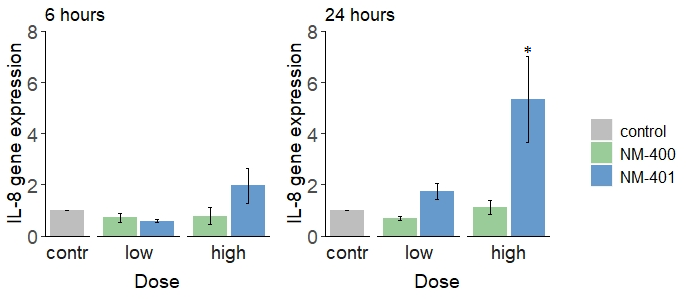 | | | | |
| --- | --- | --- | --- | --- | --- |
| **B** | 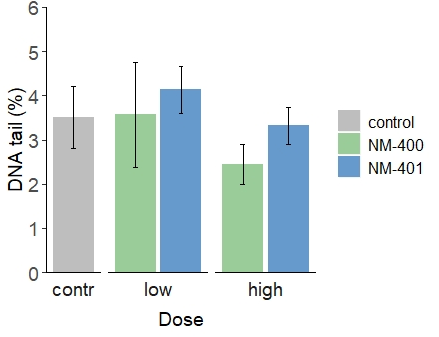 | **C** | **MWCNT** | **NM-400** | **NM-401** |
|  |  |  | **Low dose (µg)** | 2.30 ± 0.4 | 3.64 ± 1.4 |
|  |  |  | **High dose (µg)** | 9.42 ± 0.6 | 9.59 ± 0.4 |

**Figure S2.** Inflammation (A) quantified as *IL-8* gene expression from a co-culture of A549 and THP-1a cells at the apical site. Fibroblasts (WI-38) were cultured on the basolateral side. No significant changes were observed for DNA strand breaks (B) quantified as DNA tail (%) after 24. The doses were quantified with quartz crystal microbalance (C). The data are represented as mean value of 4 independent replicates ± SEM. Star represents statistical significance (p<0.05).


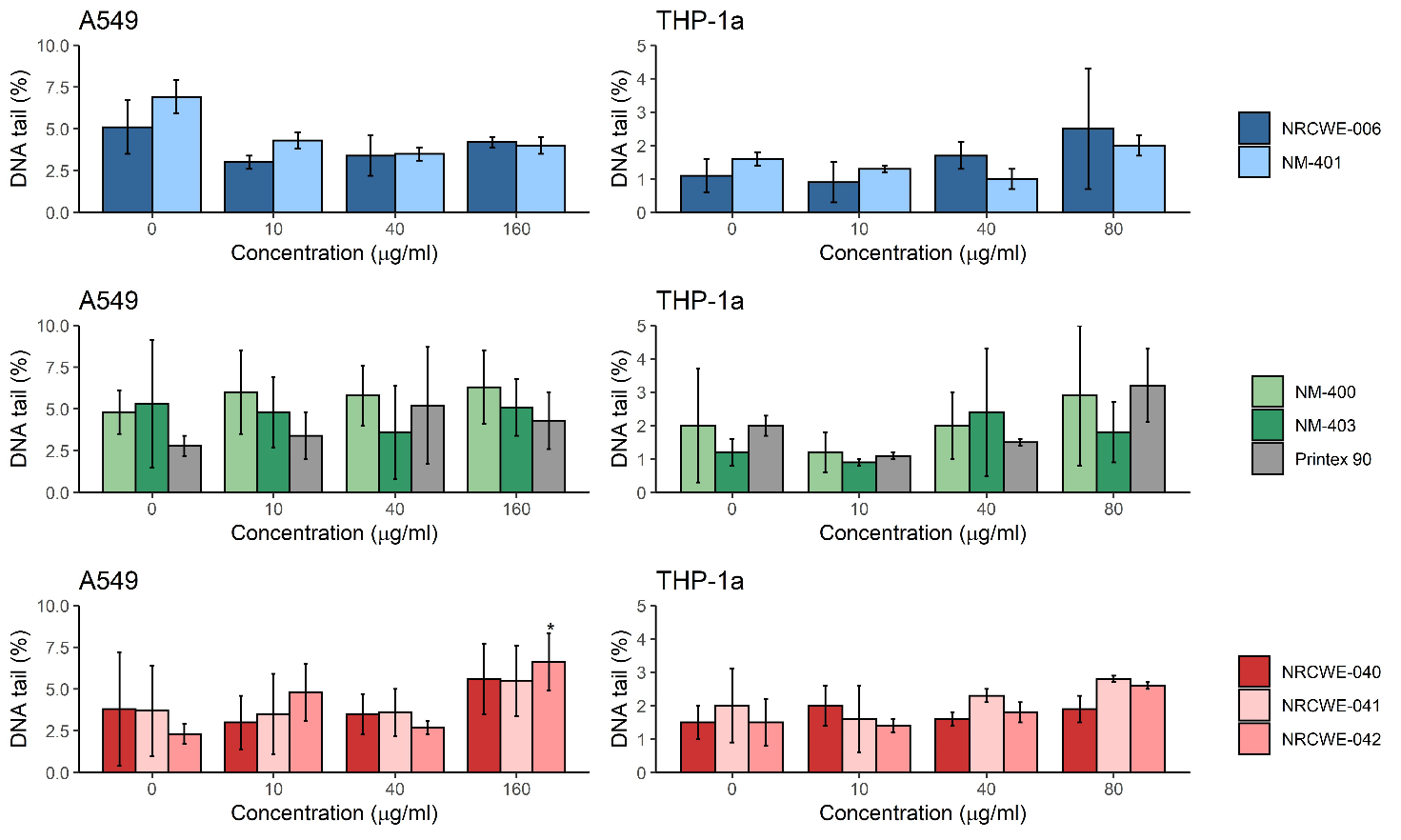


**Figure S3.** DNA damage in terms of % tail DNA in A549 and THP-1a cells exposed to NM for 6h. The values are mean ± standard deviation of minimum three independent experiments. Statistical significance assessed at p ≤ 0.05.

**
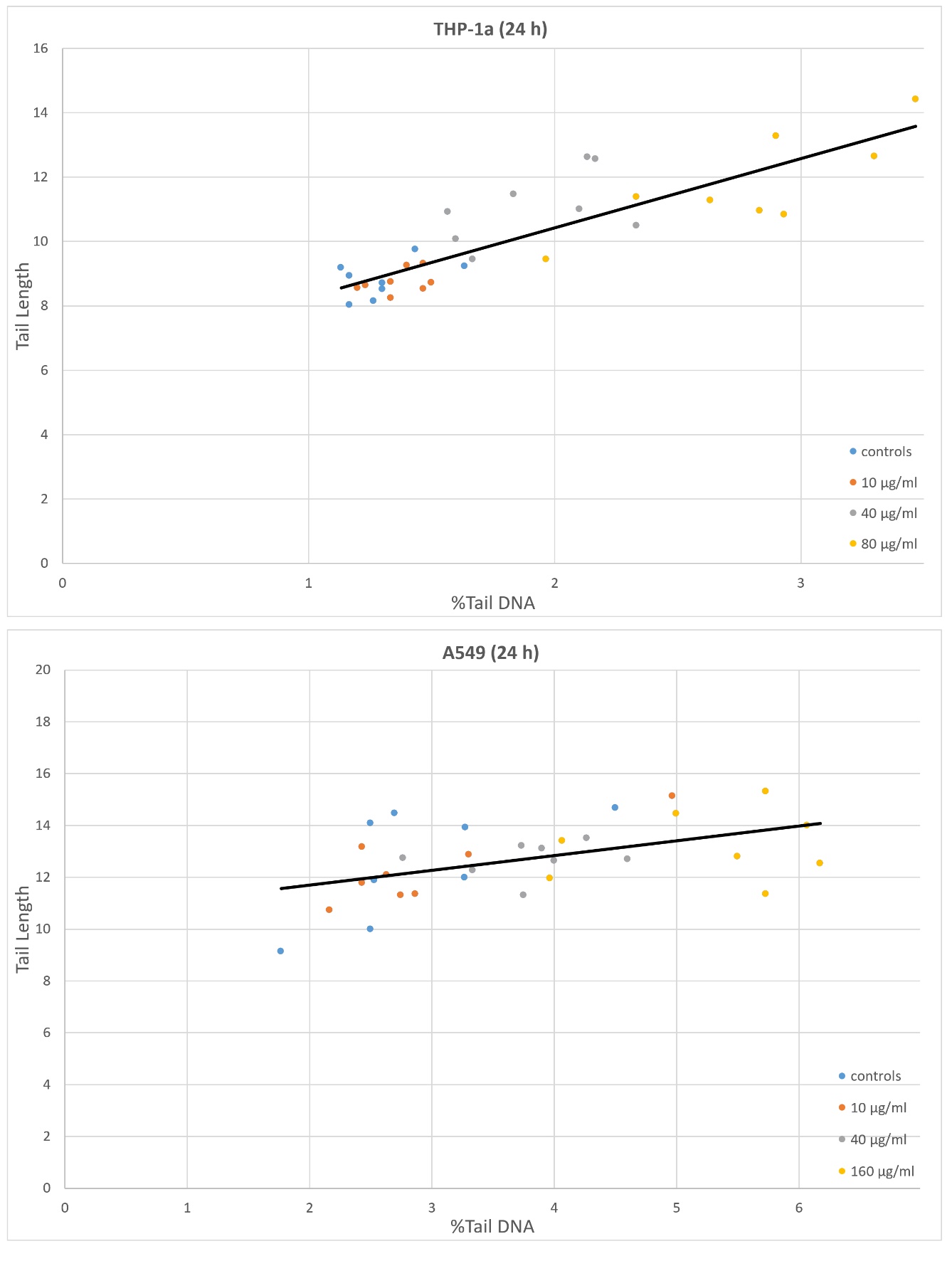
**

**Figure S4.** Illustration of the linear relationship between %Tail DNA and Tail length in the comet assay. Measuring DNA migration as tail length is likely not affected by a possible NM interference, therefore we compared tail length to % Tail DNA. In our dataset, there is a strong linear correlation THP-1a (p<0.001) and A549 (p=0.005) between % tail DNA and tail length following 24 h of exposure to all tested NM.


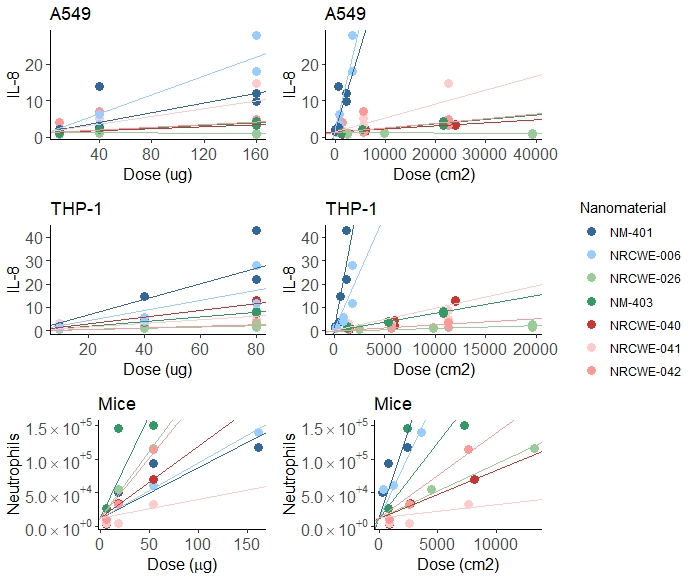


**Figure S5.** Regression of dose (in mass and surface area) with inflammation of MWCNT *in vitro* and *in vivo*. The slopes were obtained from linear regresions to assess the effect of MWCNT type and surface area on the responses (IL-8 and neutrophil influx). The *in vitro* data from A549 and THP-1a at 6 and 24 h were combined. NRCWE-026 which was tested *in vivo* corresponds to NM-400 *in vitro;* these are the same material, but produced in different batches.


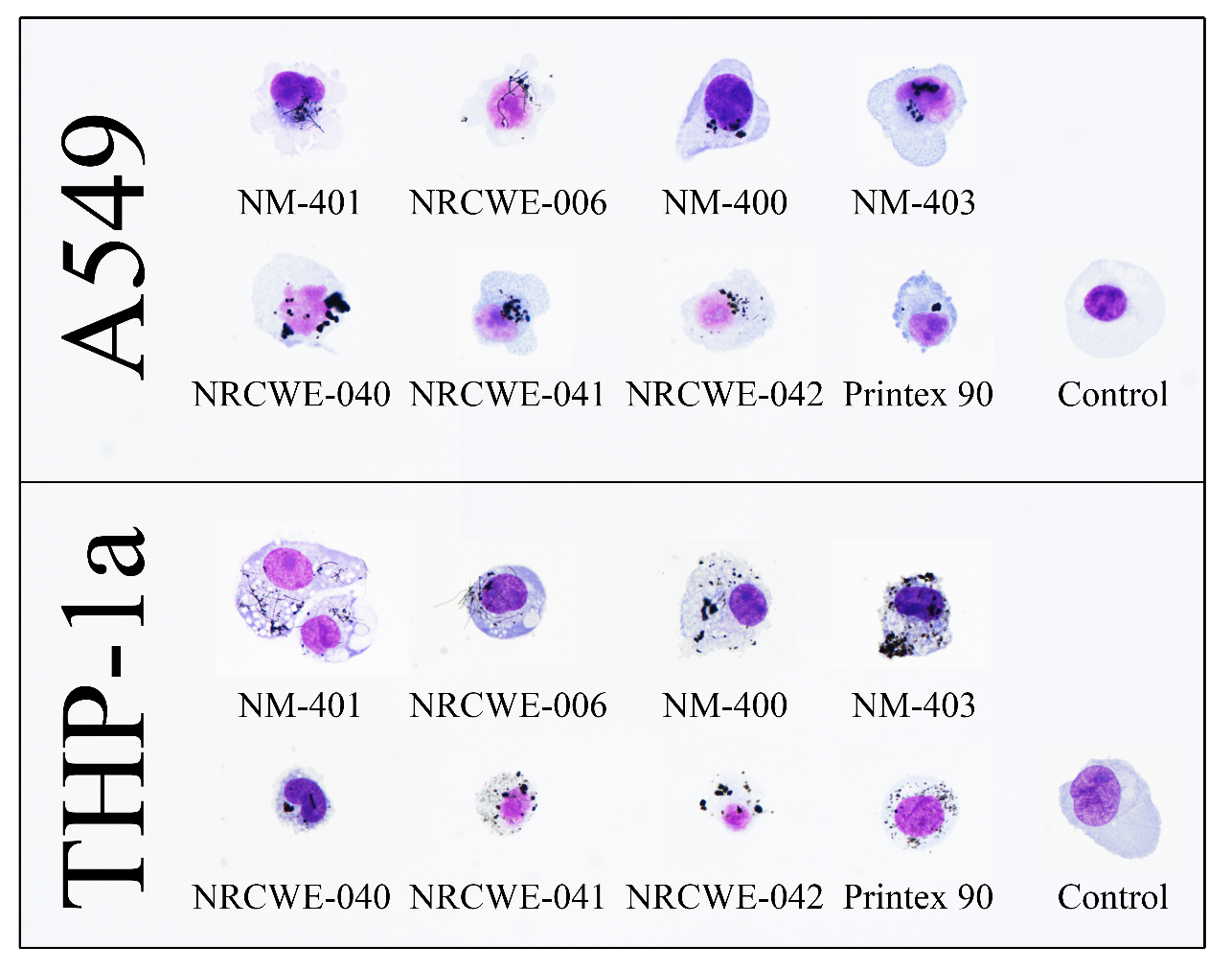


**Figure S6.** Bright field microscopy images of A549 and THP-1a cells exposed to MWCNT and carbon black. Cells were exposed to 40 µg/ml of the materials for 24h, after which trypsinized, and frozen at -80 C in freezing media containing FBS and DMSO, until fixation. Cells were stained with Geimsa and scored qualitatively at 100x magnification. The samples shown in the figure were selected as representative of the samples.
